# Development of a CD39 nanobody and its enhancement to chimeric antigen receptor T cells efficacy against ovarian cancer in preclinical studies

**DOI:** 10.7150/thno.97590

**Published:** 2024-09-30

**Authors:** Yu-Chen Zhang, Xian-Yang Li, Qi Deng, Yan-Jun Ge, Rui-Rong Yi, Hua-Jing Wang, Jian-Tao Wang, Hui Zhou, Xue-Feng Kong, Rong-Jiao Liu, Yu-Ting Zhang, Xiao-Pei Li, Xiao-Wen He, Hai-Yan Zhu

**Affiliations:** 1 Shanghai Key Laboratory of Maternal Fetal Medicine, Shanghai Institute of Maternal-Fetal Medicine and Gynecologic Oncology, Shanghai First Maternity and Infant Hospital, School of Medicine, Tongji University, Shanghai 200092, China.; 2 R&D Department, OriCell Therapeutics Co. Ltd., 1227 Zhangheng Road, Shanghai, 201203, China.; 3 Department of Gynecology, Shanghai First Maternity and Infant Hospital, Tongji University School of Medicine, Shanghai, China.; 4 Department of Obstetrics and Gynecology, The First Affiliated Hospital, Wenzhou Medical University, Wenzhou 325015, China.

**Keywords:** Ovarian cancer, CAR-T, CD39, Nanobody, Tumor microenvironment

## Abstract

**Rationale**: CD39, a key ectonucleotidase that drives adenosine production, acts as a critical immunosuppressive checkpoint in cancer. Although it has shown promise as a therapeutic target, clinical trials are demonstrating the need for more potent targeting approaches. This need is driving innovation towards the development of novel antibodies and the exploration of strategic combinations with a range of immunotherapies.

**Methods**: An anti-CD39 nanobody was screened and tested for its affinity and binding ability using biolayer interferometry, ELISA and flow cytometry. Blocking ability against soluble and membrane-bound CD39 was measured after CD39 blockade. Internalization was detected using immunofluorescence. The reversal of T-cell function by the anti-CD39 antibody was assessed by CFSE-based T-cell proliferation, CD25 expression and IFN-γ secretion. The *in vivo* function of tumor growth inhibition was further tested in a mouse model and we also tested the phenotype of immune cells after CD39 antibody administration from tumor tissue, draining lymph nodes and peripheral blood. We inserted the antibody sequence into the chimeric antigen receptor (CAR) construct to induce MSLN CAR-T cells to secret the CD39 antibody, and the efficacy was measured in xenograft models of ovarian cancer.

**Results**: We screened human CD39 antibodies using a VHH library and developed a single-epitope anti-CD39 nanobody, named huCD39 mAb, with high affinity and potent binding and blocking ability. The huCD39 mAb was internalized in a time-dependent manner. The *in vitro* study revealed that the huCD39 mAb was highly effective in enhancing T-cell proliferation and functionality. *In vivo*, the huCD39 mAb showed significant anti-tumor efficacy in an immunocompetent mouse model. Flow cytometry analysis demonstrated downregulated CD39 expression in immune cells after antibody administration. We also observed increased CD39 expression in ovarian cancer tissue and in activated CAR T cells. Subsequently, we developed a type of MSLN CAR-T cells secreting huCD39 mAb which showed effective eradication or inhibition in ovarian tumor xenografts.

**Conclusions**: A novel huCD39 mAb with strong blocking ability against human CD39 and potent inhibition of tumor growth has been developed. Furthermore, a modified huCD39 mAb-secreting CAR-T cell has been generated, exhibiting superior efficacy against ovarian cancer. This provides a promising strategy for optimizing immunotherapies in ovarian cancer and potentially other malignancies.

## Introduction

Ovarian cancer is the deadliest gynecological cancer despite the constant update of therapeutic strategies, and it accounts for 2.1% deaths from all cancers 1. Most patients are diagnosed at an advanced stage with a poor prognosis. Debulking surgery and chemotherapy are primary treatments, which can effectively prolong patients' lives, but many patients still suffer from recurrence 2,3. Immune checkpoint blockade and adoptive cell therapy are promising therapeutic strategies for tumor immunotherapy. Although the overall response rate for PD-1/PD-L1 antibodies in ovarian cancer patients has reached 4-15%, it is still moderate compare to other solid cancers 4. In this contemporary setting, the development of immune checkpoint inhibitors that target other checkpoints is of great importance.

CD39 is a recently recognized target in immunotherapies which is encoded by the *ENTPD1* gene. Extracellular ATP released under stress condition induces a pro-inflammatory microenvironment in tumors. The accumulated ATP promotes both innate and adaptive immune responses in tumor microenvironments (TME) 5-7. CD39 is a critical enzyme that catalyzes ATP into AMP, and CD73 further hydrolyses the degradation of AMP to adenosine 5-7. Adenosine is immunosuppressive which inhibits the function of T cells, DCs, and macrophages via the interaction with A2A receptor 8,9. Compared with CD8^+^ T cells in peripheral blood, tumor-specific CD8^+^ tumor-infiltrating lymphocytes (TILs) has higher expression of CD39 10,11. CD39^+^CD8^+^ T cells derived from tumors and invaded lymph nodes highly expressed inhibitory receptors, such as PD-1, Tim-3, LAG-3 and TIGIT, and are functionally exhausted 10. CD39-deficient mice exhibited delayed tumor growth and higher levels of IFN-γ from TILs compared to wild type mice 12. The above results suggested CD39 as a novel therapeutic target with significant potential.

Research on CD39 blockade has increased recently, and antibodies have shown satisfactory efficacy against solid tumors 12-19. In a phase I study, the CD39 antibody IPH5201 in combination with durvalumab achieved stable disease in 22 of 57 patients with advanced tumors. Moreover, the results from a phase I study of IPH5201 and another CD39 antibody, SRF617, suggested that anti-CD39 antibodies were well tolerated. In addition to the use of an anti-CD39 antibody as a monotherapy, combining an anti-CD39 antibody with chemotherapy or antibodies targeting CD73 or PD-1/PD-L1 in mouse xenografts has shown better efficacy 12,15,20. These studies highlight the superiority of targeting CD39; however, there remains significant potential for the development of innovative antibody strategies against this target.

Ovarian cancer is an immunogenic tumor with immunosuppressive signals in the TME 3. This characteristic may explain the poor effectiveness of immune checkpoint blockade. Chimeric antigen receptor T (CAR-T) therapy exploits tumor-associated antigen recognizing T cells, which can infiltrate the TME and exert specific cytotoxicity. Many investigated targets, such as mesothelin (MSLN), MUC-16, HER2, and folate receptor-α (αFR) are expressed in ovarian cancer tissue, which provides a basis for CAR-T development 21. Although impressive responses to CAR-T therapy have been observed in ovarian cancer, immunosuppressive TME is one of the leading causes that impaired CAR-T function, therefore, untapped opportunities to enhance the effects of CAR-T therapy in solid tumors remain 21-23. In recent years, engineered CAR-T with PD-1/PD-L1 blockade contributed to enhanced anti-tumor effects in preclinical research 24. CAR-T secreting bispecific T-cell engagers also exhibited improved efficacy against tumors 25,26. Due to the elevated expression of CD39 in ovarian cancer and the tumor-targeting capability of CAR-T therapy, the combination of CAR-T cells with an anti-CD39 antibody may yield an effective response in patients with ovarian cancer.

Our study described a single-epitope nanobody-derived anti-CD39 antibody with high affinity referred to as huCD39 mAb. The huCD39 mAb effectively promoted T cell functions *in vitro* and showed potent anti-tumor effect in mouse model. We also demonstrated that the huCD39 mAb significantly improved the anti-tumor efficacy of MSLN CAR-T cells in xenograft models of ovarian cancer. These findings provide a scientific rationale for the combination of CD39 antibodies with CAR-T therapies in clinical practice.

## Methods

### Cell lines

The cell Lines 293T and CHO and the ovarian cancer cell lines SK-OV-3 and Caov-3 were maintained in Dulbecco's modified Eagle's medium (DMEM) (11965-084, Gibco). HeLa and SK-MEL-28 cells were maintained in MEM (L540KJ, Basalmedia). Peripheral blood mononuclear cells (PBMCs) and CAR-T cells were cultured in X-VIVO medium (04-418Q, Lonza). The medium was supplemented with 10% fetal bovine serum (FBS). The cells were engineered to express MSLN or human, mouse or cynomolgus CD39 via lentiviral transduction. All cell lines were cultured at 37 °C in an atmosphere containing 5% CO_2_.

### Phage library construction

The CD39 extracellular ligand recognition domain (ECD) (ACROBiosystems, cat. no. CD9-H52H4) was used to immunize alpaca. The entire process of immunization, serum antibody titre testing, antibody purification, and VHH phage library construction was performed by Chengdu NB Biolab Co., Ltd. Briefly, PBMCs from an alpaca were isolated after three or four doses of inoculation, and RNA was extracted and transcribed into cDNA. VHH segments were amplified using PCR, and PCR products were inserted into a pComb3XSS vector. Vectors containing VHH genes were electro-transformed into competent *E. coli* TG1 cells. The capacity and diversity of the VHH phage library were tested.

### Panning of VHH antibodies against human CD39

Panning of the VHH phage library was performed as described previously 27. Briefly, 1-10 μg/mL human CD39 ECD protein was used to coat 96-well plates at 4 °C overnight, and the plates were blocked with 5% non-fat milk containing human IgG1-FC for 2 h at room temperature. The phage library was used and incubated at room temperature for 1-2 h. Unbound phages were washed away using PBS + 0.05% Tween-20 and phages displaying VHHs targeting CD39 were collected. After each round of library panning, enriched phages were amplified and titrated. ELISA of the supernatant of the phages after amplification and DNA sequencing of several single colonies were used to determine the specificity and diversity of the phages after each round of panning.

After two to three rounds of panning, individual clones were tested via monoclonal ELISA against human CD39 or the human IgG-FC protein for their specific binding to human CD39, positive phages were selected and their DNA was sequenced.

### Construction, expression and purification of antibodies

DNA fragments of phages with unique CDR3 regions were cloned and inserted into the pcDNA3.4-TOPO vector containing DNA fragments of human IgG1 Fc. The plasmids were transfected into Expi293 cells with PEI for 5 days. Supernatants containing CD39-targeting antibodies were collected and purified as described previously 28. SDS-PAGE analysis was used to analyze the purity of antibodies, antibodies boiled with or without β-mercaptoethanol were electrophoresed in a 12.5% sodium dodecyl sulfate-polyacrylamide gel and stained using Coomassie brilliant blue.

### Biolayer interferometry (BLI)

The binding kinetics of antibodies to CD39 proteins were determined via BLI on an Octet^RED^ 384 instrument. AHC biosensors were hydrated in buffer containing 0.1% BSA and 0.05% Tween-20 for more than 20 min. Serial dilutions of human CD39 proteins (top 100 nM with 2-fold dilution) and antibodies (100 nM) were prepared. After 60 s baseline, the antibodies were loaded for 120 s, followed by 180 s of association and 360 s of dissociation. The biosensors were regenerated in 10 mM glycine. The data were processed and analyzed using Octet® Analysis Studio Software.

### CD39 enzyme-linked immunosorbent assay (ELISA)

To detect the binding of antibodies to the CD39 protein, 2 μg/mL human/mouse/cynomolgus CD39 or IgG-FC proteins were coated on a 384-well plate overnight at 4 °C and then blocked with 5% non-fat milk for 2 h at room temperature. After washing with PBS + 0.05% Tween-20 three times, antibodies diluted in PBS were added, and the samples were incubated for 1 h at room temperature. After three washes, 1:10000 diluted anti-human IgG-Fc (HRP) antibody (Abcam, ab97225) was added, and the mixture was incubated at room temperature for 30 min. After washing with PBS + 0.05% Tween-20 for seven times, 25 μL of tetramethylbenzidine (Thermo Fisher Scientific, 002023) was added, and the mixture was incubated at room temperature in the dark. A total of 25 μL of ELISA stopping solution (Sangon Biotech, E661006-0100) was added and the absorbance was read at 450 nm on an Epoch ELISA reader (BioTek).

### Binding capacity of CD39 antibodies to cells

Serial dilutions of antibodies were prepared in PBS containing 0.01% BSA. The antibodies were incubated with the digested cells at 4 °C for 60 min, and the unbound antibodies were removed by washing with PBS. A 1:200 dilution of anti-human IgG-Fc (DyLight 650) (Abcam, ab98593) secondary antibody was added, and the mixture was incubated at 4 °C for 30 min. After washing with PBS, the cells were resuspended and detected using the IntelliCyt IQue screener.

### Antibody-mediated inhibition of enzymatic activity of soluble and membrane-bound CD39

CD39 enzyme activity was measured using a CellTiter-Glo^®^ Luminescent Cell Viability Assay (G7571, Promega) which detected the remaining ATP in the system.

For soluble CD39 activity analysis, a final concentration of 50 nM human CD39 ECD (ACROBiosystems, cat. no. CD9-H52H4) or mouse CD39 (ACROBiosystems, cat. no. CD9-M52H3) or cynomolgus CD39 (ACROBiosystems, cat. no. CD9-C5PH3) was mixed with serially diluted antibodies and incubated in assay buffer (25 mM Tris, 5 mM MgCl_2_, 0.005% Tween-20, pH = 7.5) at 37 °C for 120 min. ATP was added to a final concentration of 500 μM and incubated for 60 min, CellTiter-Glo reagent was added at a ratio of 1:1 and luminescence was recorded using the SpectraMax iD3 (Molecular Devices).

For membrane-bound CD39 activity analysis, human CD39-expressing SK-MEL-28 cell lines and 293T cells overexpressing mouse Cd39 or cynomolgus CD39 were used. The cells were treated with serial dilutions of antibodies in assay buffer at 37 °C for 30 min, ATP was added to a final concentration of 150 μM and the mixture was incubated for 60 min. The supernatant was collected followed by 500 × g centrifugation at room temperature for 5 min. CellTiter-Glo reagent was added at a ratio of 1:1 and luminescence was recorded. The enzyme inhibition rate was calculated with the following formula: ((CD39 protein/CD39 positive cells + Ab + ATP) - (CD39 protein/CD39 positive cells + ATP))/(ATP - (CD39 protein/CD39 positive cells + ATP)) × 100%.

### Functional verification of the effect of the anti-CD39 antibody on PBMC-derived T cells

PBMCs were isolated using Ficoll-Paque PREMIUN (Cytiva) according to the manufacturer's instructions. After washing with saline solution one time, the PBMCs were counted and resuspended in saline solution to a final concentration of 5-10E6/mL. The resuspended PBMCs were stained with CFSE (C34570, Invitrogen) at a final concentration of 1 μM according to the manufacturer's instructions. PBMCs were stimulated with CD3/CD28 Dynabeads according to the manufacturer's instructions (40203D, Gibco). A series of dilutions of antibodies were incubated with 5E5 PBMCs per well in 24-well plates at 37 °C. After 30 min, ATP was applied at a final concentration of 250 μM. After 4 days, the cells were counted using a Nexcelom Cellometer. The supernatants were collected and the level of IFN-γ was evaluated using a Cytometric Bead Array Kit (558269, BD). The expression of CD4, CD8, and CD25 was tested using flow cytometry. ATP level from supernatant was detected using a CellTiter-Glo^®^ Luminescent Cell Viability Assay.

### Internalization of the anti-CD39 antibody in CD39 overexpressing cell lines

Internalization was performed as previously described 29. Briefly, CHO-h39-1 and SK-OV-3-MSLN^hi^-CD39^hi^ cells overexpressing CD39 were digested and incubated at 4 °C for 30 min, and 20 μg/mL anti-CD39 antibody or human IgG-FC was added and incubated at 4 °C for 1 h. The stained cells were washed three times with cold PBS, and a 1:200 dilution of anti-human IgG-Fc (DyLight 650) secondary antibody was added and incubated at 4 °C for 30 min. After another round of washing with cold PBS, the cells were resuspended in PBS and incubated at 37 °C to induce internalization for the indicated times. Then, 0.4% paraformaldehyde was used to fix the cells, and the cell nuclei were stained with 2 μg/mL DAPI (D9542, Sigma). Caov-3 cells were used as a negative control. Images were taken using a Leica Microsystems confocal fluorescence imaging microscope.

### Molecular modelling and cross-competition assay

The CD39 protein format was downloaded from the AlphaFold Protein Structure Database. The structure of huCD39 mAb was predicted using AlphaFold2 in the Google Colab notebook 30. Protein-protein docking was performed using the Cluspro web server in antibody mode 31-35. The complexes were visualized using the Pymol Molecular Graphics System, version 2.5.0.

A cross-competition assay was performed via BLI on an Octet^RED^ 384 instrument. The AHC biosensors were hydrated, 100 nM CD39 mAb was loaded for 180 s, and 50 μg/mL IgG1-FC was added to block the remaining AHC biosensors. CD39 mAb or H5L5 antibody (1 μM) was premixed with 100 nM CD39 His protein at 4 °C overnight, and the mixtures were loaded for 300 s.

### *In vivo* anti-tumor efficacy of the anti-CD39 antibody in a mouse xenograft model

C57BL/6-*Nt5e*^tm1(*hNT5E*)^*Entpd1*^tm3(*hENTPD1*)^/Bcgen mice (111855, Biocytogen) were inoculated with 1E5 MC38 cells subcutaneously. The mice were randomly divided into 5 groups when the tumor area reached approximately 10-20 mm^2^. All of the mice were treated intraperitoneally (i.p.) with the PBS vehicle or various antibodies every 3 days for 6 doses. Three additional doses of huCD39 mAb were administered every 3 days starting from day 25 until euthanasia. Tumor volume and body weight were measured and recorded three times per week. Tumor tissues, blood, and draining lymph nodes in the huCD39 mAb and vehicle groups were harvested for further analyses.

### Flow cytometry

The tumor tissue was dissected and digested using DNase I (C5138-100MG, Sigma) and collagenase IV (D5025-150KU, Sigma) at 37 °C. After filtering through a 70-μm nylon cell strainer, the cells were collected and centrifuged for 5 min at 1200 rpm. Staining was performed at 4 °C for 30 min and the samples were immediately analyzed in a BD LSRFortessa flow cytometer.

Immune cells in the mouse model were identified using anti-mouse CD335 (NKp46) (137612, BioLegend, RRID: AB_2563104), anti-mouse CD19 (115543, BioLegend, RRID: AB_11218994), anti-human CD39 (328212, BioLegend, RRID: AB_2099950), anti-mouse F4/80 (743282, BD, RRID: AB_2741400), anti-mouse Ly-6G/Ly-6C (Gr-1) (108443, BioLegend, RRID: AB_2562549), anti-mouse CD11b (5577657, BD, RRID: AB_396772), anti-mouse CD25 (102036, BioLegend, RRID: AB_2563059), anti-mouse CD45 (103138, BioLegend, RRID: AB_2561392), anti-mouse CD3 (100212, Biolegend, RRID: AB_389301), anti-mouse CD4 (100412, BioLegend, RRID: AB_312697), and anti-mouse CD8a (100707, BioLegend, RRID: AB_312747). Dead cells were excluded using 7-AAD (559925, BD, RRID: AB_2869266). Absolute cell counts were quantified using Precision Count Beads (424902, BioLegend).

The expression of human CD39 on CHO-h39-1, 293T-c39-1, Caov-3, SK-OV-3 and SK-OV-3-MSLN^hi^-CD39^hi^ cells was tested with an anti-human CD39 antibody (560239, BD, RRID: AB_1645459). The expression of mouse CD39 on 293T-m39-9 cells was tested with an anti-mouse CD39 antibody (143803, BioLegend, RRID: AB_11218603). The expression of MSLN and CD19 on tumor cell lines was tested by incubating cells with 3 μg/mL anti-MSLN-IgG-FC or anti-CD19-IgG-FC protein (the antibody used to develop the CAR construct) at 4 °C for 60 min. After washing with PBS, a 1:200 dilution of anti-human IgG-Fc (DyLight 650) secondary antibody was added at 4 °C for 30 min. The expression of CD39 and CD73 on PBMCs was determined with anti-human CD39 antibody (560239, BD, RRID: AB_1645459) and anti-human CD73 antibody (560847, BD, RRID: AB_10612019).

CAR expression was measured using flow cytometry. 1E6 CAR-T cells were collected and incubated with 5 mg/kg MSLN-his protein or CD19-his protein for 60 min. After washing with PBS, a 1:200 dilution of DyLight® 488 Anti-6X His tag® antibody (ab117512, Abcam) was added with other antibodies, including APC-H7 Anti-Human CD4 (560158, BD, RRID: AB_1645478), PE/Cyanine7 anti-human CD8 (344712, BioLegend, RRID: AB_2044008), BV510 Anti-Human CD45RO (563215, BD, RRID: AB_2738074) and PE anti-human CCR7 (130-119-583, Miltenyi), and incubated for 30 min, 7-AAD was added 10 min before analysis.

### Bioinformatic analysis

Publicly available datasets were downloaded from the Gene Expression Omnibus (GEO) database under accession numbers GSE184880, GSE160311, and GSE231310 36-38. The details of the studies analyzed are as follows—the GSE184880 dataset analyzed single-cell transcriptomes from seven high-grade serous ovarian cancer (HGSOC) samples and five non-malignant ovarian tissues. The GSE160311 dataset contained RNA sequencing data of EGFR-28zCAR-T cells after co-cultured with EGFR^+^ A549 cells for 0, 6, 24, and 72 hours. The GSE231310 dataset investigated the transcriptome of CD19^+^ CAR-T stimulated with CD19^+^ Nalm6 cells for different time periods.

The Seurat package 3.1.4 (https://satijalab.org/seurat/) was used for normalization, regression, batch effect removal, and clustering. Pseudotime trajectory analysis was performed using Monocle 2.0 39-41. Differentially expressed genes in the RNA sequencing datasets were identified via using DEseq2 42. Visualizations such as heatmap and boxplots were generated using R package ggplot2 and ComplexHeatmap. The data of the ovarian cancer cohort (n = 421) from The Cancer Genome Atlas (TCGA) database were downloaded from the Genomic Data Commons Data Portal. The general immune scores were calculated using Consensus^TME^ and ESTIMATE, and the Spearman correlation coefficient was used to evaluate *ENTPD1 (CD39)* expression and general immune scores in the TCGA ovarian cancer cohort 43,44.

### Construction of CARs

Anti-CD39 mAb constructs were cloned and inserted into a third-generation lentiviral plasmid backbone. MSLN CAR constructs contained a VHH sequence targeting the MSLN protein, a CD8 transmembrane domain in tandem with a 4-1BB domain and a CD3ζ signaling domain. Antibody sequences against CD39 were flanked by an Igκ leader peptide and a His-tag element, and inserted downstream of the CAR construct with a T2A self-cleaved sequence. CD19 CAR constructs contained a VHH sequence targeting the CD19 protein.

### CAR-T-cell production

Healthy donor-derived PBMCs were used to isolate T cells using CD3 microbeads (130-050-101, Miltenyi) according to the manufacturer's instructions. Isolated T cells were activated with CD3/CD28 Dynabeads for 24 h (40203D, Thermo Fisher Scientific). CAR-T were generated via lentiviral transduction supplemented with polybrene, then horizontally centrifuged at 1200 rpm for 1 h. Lentiviruses were removed after 24 h and replaced with fresh X-VIVO medium supplemented with FBS. CAR expression and cytotoxicity were measured 7 days after infection, and the cells were expanded *in vitro* for 9 days after infection until harvested for freezing. Remains of the CAR-T were tested on day 9 for the CAR-positive ratio. Frozen CAR-T cells were thawed, resuspended in PBS and counted for cell viability before* in vivo* infusion.

### Verification of antibody secretion using T-cell supernatant

The supernatant of the CAR-T cells used for the animal experiments was collected, filtered, and purified via binding NI-NTA-Agarose, and the purified protein was eluted with imidazole and dialyzed against PBS.

The purified proteins were separated via 10% sodium dodecyl sulph-PAGE (SDS‒PAGE) and transferred onto a polyvinylidene difluoride membrane. After blocking with 5% non-fat milk, the membrane was incubated with an anti-His antibody (ab1187, Abcam) at 4 °C overnight. The protein bands were visualized via chemiluminescence on a Tanon 4600 instrument.

### *In vitro* evaluation of antibody-secreting MSLN CAR-T cells

Cytotoxicity of CAR-T cells was measured by coculturing of 2E4 or 6.7E3 CAR-T cells with 6E4 SK-OV-3-MSLN^hi^-CD39^hi^ ovarian cells. After 16-24 h, the cells were centrifuged at 500 × g for 5 min, and half of the supernatant was removed. Cytotoxicity was measured using the ONE-Glo™ Luciferase Assay System (E6120, Promega).

CAR-T-cell proliferation was tested by the coculture of 3E5 CAR-T cells with 3E5 SK-OV-3-MSLN^hi^-CD39^hi^ ovarian cells. Four days after coculture, the CAR-T cells were counted using AO/PI dyes on a Nexcelom Cellometer and some of the CAR-T cells were used to measure the CAR-positive proportion via flow cytometry. Another round of coculture was continued with 3E5 CAR-T cells and 3E5 SK-OV-3-MSLN^hi^-CD39^hi^ ovarian cells. After three rounds of coculture, the cumulative fold change was calculated.

### *In vivo* anti-tumor efficacy of MSLN CAR-T cells secreting the CD39 antibody

B-NDG mice (110586, Biocytogen) were inoculated with 3E6 SK-OV-3-MSLN^hi^-CD39^hi^ cells subcutaneously. The mice were randomly divided when the average tumor volume reached 75-80 mm^3^. The mice were treated intravenously (i.v.) with 1E7 untreated T cells, MSLN CAR-T cells or MSLN CAR-T cells secreting huCD39 mAb. Mice in another group received MSLN CAR-T cells intravenously (i.v.) and the huCD39 mAb was injected intraperitoneally (i.p.). The antibodies were administered every three days for a total of eight doses. The mice were sacrificed when the tumor reached 2000 mm^3^. Tumor volume and body weight were measured and recorded three times per week. Peripheral blood was collected weekly, and CAR-T cell levels were measured via flow cytometry, and the levels of the cytokines IL-2 and IFN-γ were detected using the Cytometric Bead Array Kit. Organs were collected after euthanasia and placed in 10% formalin before embedding in paraffin. After embedding, the tissue was sectioned into 5-μm paraffin sections, and HE staining was performed via deparaffinization, rehydration, staining, dehydration and sealing. Images were captured using a scanning machine or a Leica DMi8 microscope.

B-NDG mice (110586, Biocytogen) were inoculated with 4E6 SK-OV-3-MSLN^hi^-CD39^hi^ cells subcutaneously. The mice were randomly divided when the tumor volume reached 115-135 mm^3^ on average. The mice were treated intravenously (i.v.) with 6E6 CD19 CAR-T cells, CD19 CAR-T cells secreting huCD39 mAb, MSLN CAR-T cells or MSLN CAR-T cells secreting huCD39 mAb. The mice were sacrificed when the tumor reached 2000 mm^3^. Three mice in the MSLN CAR-T-cell group were euthanized on day 20 after CAR-T-cell infusion because the tumor had broken poorly and the wounds had not healed. Tumor volume and body weight were measured and recorded three times per week. Peripheral blood was collected weekly, CAR-T cell levels were measured via flow cytometry, and the levels of cytokines IL-2 and IFN-γ were detected using the Cytometric Bead Array Kit. At the endpoint of the experiment, all mice were anaesthetized and injected with 150 mg/kg D-luciferin i.p., and the tumors were recorded via bioluminescence imaging. Three mice in CD19-huCD39 mAb CAR-T group died after anesthesia. Blood serum enzymatic tests were performed at the Shanghai Institute of Materia Medica Chinese Academy of Sciences.

### Statistical analysis

GraphPad Prism 8 was used for statistical analyze. For nonparametric data, the Kruskal-Wallis test was used to compare three or more groups, followed by Dunn's multiple comparisons test. Two-way ANOVA was used for the animal experiments followed by Tukey's multiple comparisons test. Differences between two groups were compared using the Mann Whitney test.

### Ethics statement

Fresh PBMCs from non-malignant patients were collected from the Shanghai First Maternity and Infant Hospital. Ethical approval was obtained from the Ethics Committee of the Obstetrics and Gynecology Hospital of Tongii University (KS23354). The animal studies were approved by the Laboratory Animal Research Center, Tongji University (TJBG14724101) and Youshu LIFE Technology (Shanghai) Co., LTD (Ys-m202401001) and the Shanghai Model Organisms Center, Inc. (2024-0027).

## Results

### Generation of a VHH nanobody targeting human CD39

**Figure 1:**
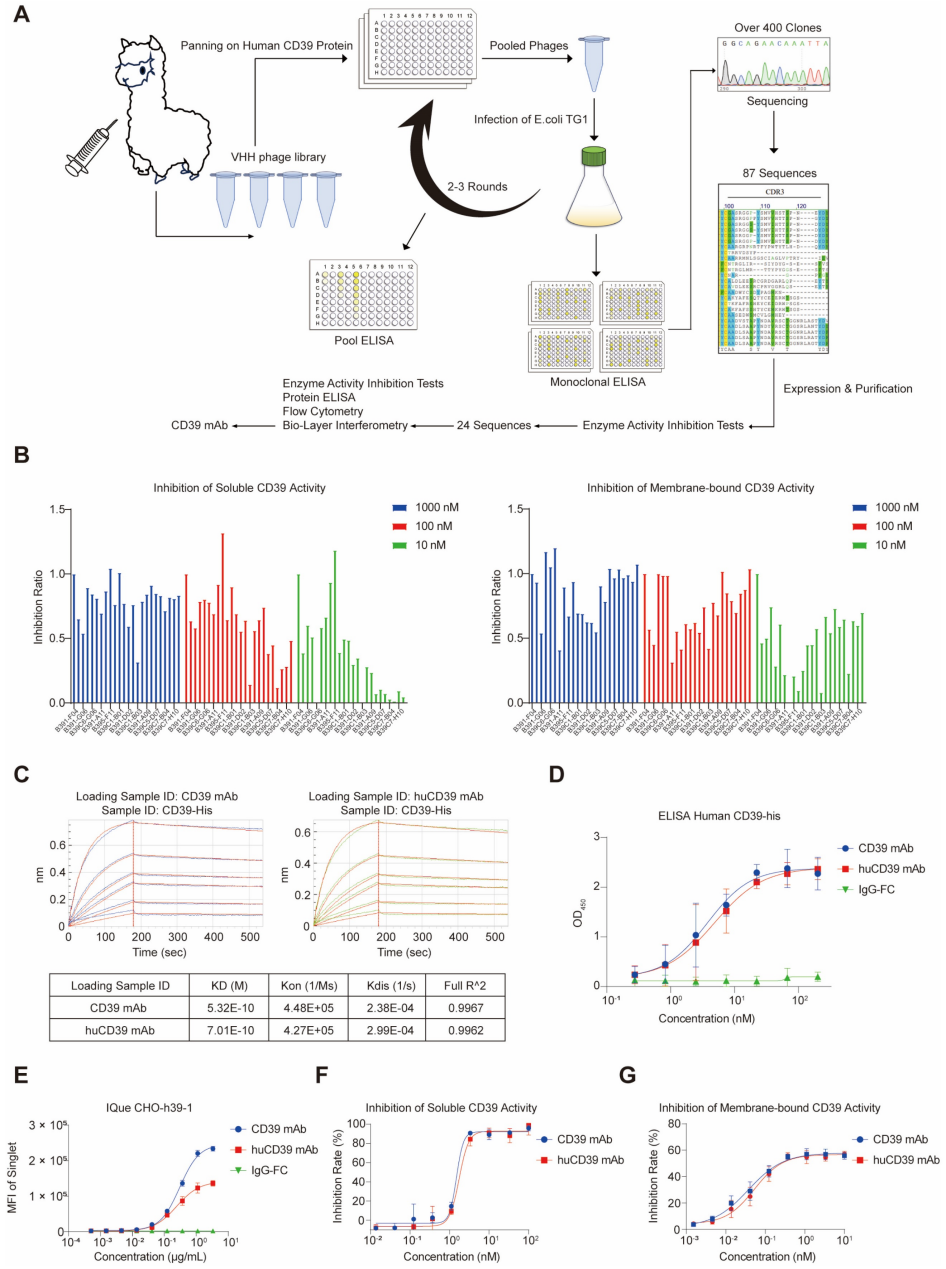
** Immunization of alpaca produced an anti-CD39 nanobody with high affinity and potent blocking ability.** (A) Schematic diagram of antibody screening against human CD39. (B) Blocking of enzymatic activity on soluble and membrane-bound CD39 were further tested on 24 selected antibodies. (C) CD39 mAb and huCD39 mAb bind to CD39-his protein as measured via biolayer interferometry (BLI). (D) Binding of CD39 mAb and huCD39 mAb to CD39-his protein was measured via ELISA. (E) IQue-based flow cytometry showed binding of CD39 mAb and huCD39 mAb to human CD39 on cell membrane. (F-G) Soluble (F) or membrane-bound CD39 on SK-MEL-28 cells (a CD39 positive cell line) (G) were blocked with indicated concentration of antibodies. Data are shown as mean ± SD (n = 3).

Current CD39 antibodies are typically based on the human immunoglobulin G format, which consists of two heavy chains and two light chains. To create smaller, high-affinity antibodies with the greater tissue penetration, we immunized an alpaca with the human CD39 extracellular ligand recognition domain (ECD) protein (Figure 1A). Following four doses of immunization, we constructed a VHH phage library against human CD39, which had a size of 2.96 × 10^13^ and diversity of 1.16 × 10^9^. Two to three rounds of screening of the VHH phage library yielded over 400 potential clones with different sequences (Figure S1A). From this library, we selected 87 unique sequences based on the CDR3 region and inserted these VHH genes into a pcDNA3.4-TOPO vector containing human IgG1 Fc DNA fragments. Following expression and purification, we evaluated the ability of these 87 antibodies to inhibit CD39 enzyme activity in soluble and membrane-bound forms. Among all of the antibodies tested, one (B391-F04) exhibited the most effective inhibitory effect on soluble and membrane-bound CD39 (Figure S1B).

Further biochemical characterization was performed on the 24 antibodies that showed satisfactory blocking ability (Figure 1B). The ability of the antibodies to bind to the human CD39 ECD protein was examined using ELISA. The results revealed that all 24 antibodies bound to soluble CD39 proteins, with the B391-F04 antibody having the lowest EC50 (Figure S1C and Table S1). BLI analysis was performed to determine the binding affinity of the antibody for CD39. The results showed that these antibodies bound to CD39 with nanomolar affinity (K_D_ = 0.1-10 nM) (Table S2). Binding specificity and affinity were further tested in CHO cells and human *CD39*-overexpressing CHO cells (Figure S1D). The antibodies exhibited different binding affinities to *CD39*-overexpressing CHO cells, and limited non-specific binding to CHO cells (Figures S2A-B). B391-F04 had an extremely low EC50 value of 0.31 μg/mL.

### Humanized CD39 mAb retained its affinity and blocking ability to CD39

We selected B391-F04 for further study (termed the CD39 mAb) because it outperformed the other candidates in multiple aspects. To humanize the antibody, we mutated eight amino acids (Q1E, L2V, A75S, N85S, K87R, P88A, G92A and Q110L) to resemble the DP-47 sequence based on prior knowledge 45. To prevent any antibody-dependent cellular cytotoxicity (ADCC) or complement-dependent cytotoxicity (CDC) activities, we fused the humanized CD39 mAb (termed huCD39 mAb) to a modified IgG4 Fc domain lacking the CH1 region and containing an S108P mutation in the hinge region. Purified antibodies were confirmed via SDS-PAGE (Figure S1E). BLI and ELISAs demonstrated that both antibodies bound human CD39 with comparable affinities (Figures 1C-D). The binding affinity of the huCD39 mAb to membrane-bound CD39 was similar to the original CD39 mAb, and the huCD39 mAb retained its ability to inhibit CD39 enzyme activity (Figures 1E-G and Figure S4A). Another CD39 antibody H5L5 also showed great blocking ability for CD39, and huCD39 mAb was more potent against soluble CD39 than H5L5 (Figures S1F-G). These results suggested that the huCD39 mAb maintained its binding affinity and blocking ability after humanization.

### HuCD39 mAb was internalized

**Figure 2:**
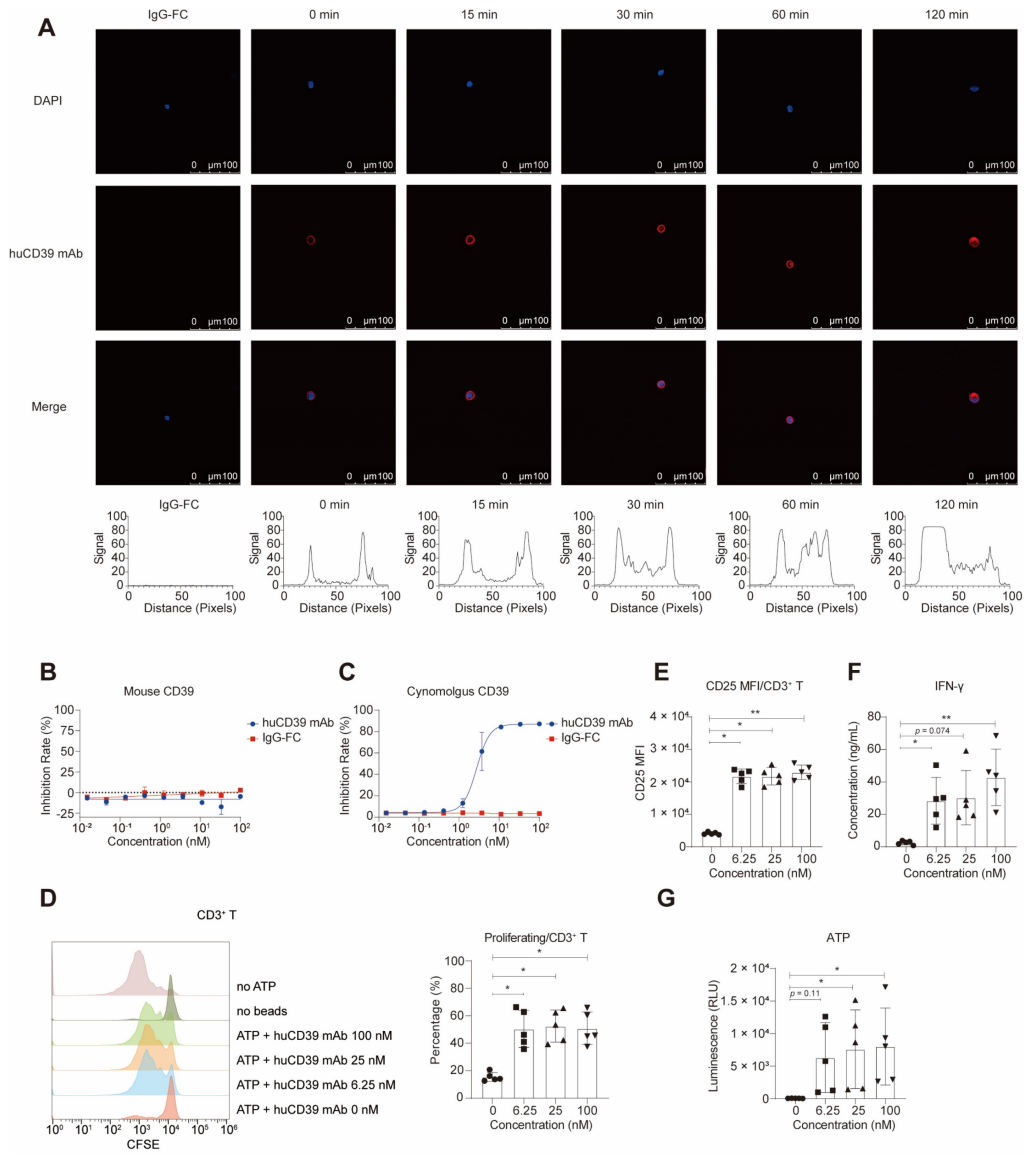
** HuCD39 mAb induced internalization, blocked cynomolgus CD39 and reversed adenosine caused T cell dysfunction.** (A) Internalization was induced for indicated time, cell nuclei (blue) and huCD39 mAb antibody (red) were shown via immunofluorescence staining. Red signal on one representative cell at each indicated time was measured using ImageJ, signals detected on both ends were on the cell membrane and signals in the middle were in the cytosol. Data is representative of at least three biologically independent experiments. (B-C) Blocking of soluble mouse (B) and cynomolgus CD39 (C) enzymatic activity was detected. Data are shown as mean ± SD (n = 3). (D-G) PBMC derived T cells were activated with anti-CD3/CD28 Dynabeads and then incubated with antibodies for 30 min at 37 °C, ATP was added and cells were cultured for 4 days. Proliferation measured via CFSE dilution assay through flow cytometry showed increased proliferation after huCD39 mAb treatment (D). CD25 MFI assessed via flow cytometry showed elevated activation after huCD39 mAb treatment (E). IFN-γ level in supernatants evaluated via Cytometric Bead Array kit showed increased cytokine secretion after huCD39 mAb treatment (F). ATP level in supernatants of PBMCs was measured (G). *, P < 0.05; **, P < 0.01 by one-way ANOVA test, followed by Dunn's multiple comparisons test. The data presented is representative of samples collected from five distinct donors.

To investigate the potential role of antibody-induced internalization in the downregulation of antigens, we determined whether the CD39 antibody induced this process in two CD39-overexpressing cell lines, SK-OV-3-MSLN^hi^-CD39^hi^ cells and CHO-h39-1 (Figure S3D). Our results showed that after 30 minutes of incubation, the DyLight^TM^ 650-labelled huCD39 mAb and H5L5 began to be internalized into the vesicle of SK-OV-3-MSLN^hi^-CD39^hi^ cells, with the most significant internalization occurring after 60 minutes of incubation. The antibody, indicated by red light, aggregated in the vesicle, and the red light on the membrane became weak (Figure 2A and Figure S3A). The results revealed that the huCD39 mAb induced internalization in a time-dependent manner, which indicated successful binding to its target. The internalization was also detected in the CHO-h39-1 cell line (Figures S3B-C). Caov-3 cells which do not express human CD39 was used as negative control, and no signal was detected (Figures S3E-F).

### HuCD39 mAb bound to cynomolgus CD39 but not to mouse Cd39

To determine whether these antibodies could be directly applied to pharmacokinetic evaluation assays in rodent and non-rodent species, we constructed 293T cells overexpressing mouse Cd39 and cynomolgus CD39 respectively (Figure S1D). We tested the binding of huCD39 mAb to proteins and 293T cells overexpressing mouse Cd39 and cynomolgus CD39. The results showed that the huCD39 mAb bound to cynomolgus-derived CD39 proteins that were overexpressed in 293T cells (Figures S4B-D). However, this antibody does not bind to mouse Cd39 overexpressed on the cell membrane. Enzyme activity inhibition assays revealed that huCD39 mAb only blocked the enzyme activities of cynomolgus CD39 but not mouse Cd39 (Figures 2B-C and Figures S4E-F). Due to this lack of inhibitory activity on mouse Cd39, we tested the *in vivo* anti-tumor efficacy of our antibodies in human *CD39* knock-in mice.

### The CD39 mAb shared similar epitope on the CD39 protein with H5L5

According to patents of other available anti-CD39 antibodies, several antibodies bind to similar epitopes of the CD39 protein. Therefore, we investigated the possible binding epitopes of huCD39 mAbs. The 3D Structures of huCD39 mAb were obtained via online Alphafold2 colab 30, the structure of theCD39 protein was downloaded from the Uniprot AlphaFoldDB database 46, and cluspro was used to dock the two proteins. GLU-142 and GLU-143, which are also the binding epitopes of other anti-CD39 antibodies (including H5L5), were involved in the binding interaction, as indicated (Figure S4G). To test this hypothesis, we first loaded a CD39 mAb on the AHC biosensor, and blocked the biosensor with IgG-FC. The CD39 protein premixed with various antibodies (excessive) were added to the system. The CD39 protein alone bound the CD39 mAb on the AHC biosensor, and the CD39 + CD39 mAb pre-mixture failed to bind the CD39 mAb due to the already saturated epitope on CD39. To our surprise, the CD39 + H5L5 pre-mixture also failed to bind the CD39 mAb, which indicated that the CD39 mAb shared similar epitopes with H5L5 (Figure S4H). These results revealed that the huCD39 mAb likely shared binding epitopes with other anti-CD39 antibodies, including H5L5, as supported by the structural analysis and experimental evidence demonstrating competitive binding of the CD39 protein to the huCD39 mAb and H5L5.

### Reversion of T-cell dysfunction by huCD39 mAb *in vitro*

To evaluate the impact of huCD39 mAb on T-cell proliferation, we cultured CFSE-labelled PBMCs in medium containing ATP and varying concentrations of anti-CD39 antibodies. Because CD39 and CD73 are abundant on B cells, monocytes, and other immune cells, we hypothesized that ATP in the system would be rapidly degraded into adenosine 47. We also detected expression of CD39 and CD73 in PBMCs (Figure S5A). Consistently, we observed that medium containing only ATP had a noticeable inhibitory effect on T-cell proliferation (Figure 2D). In the presence of huCD39 mAb, the inhibitory effect on T-cell proliferation was reversed. This effect was observed for CD4^+^ T and CD8^+^ T cells (Figures S5B-C). We detected increases in the CD25 MFI and IFN-γ levels following CD39 blockade (Figures 2E-F and Figures S5D-F). The efficacy of the various antibody concentrations did not differ, possibly due to a saturating dose. We also detected increased level of ATP in supernatants of PBMCs after CD39 blockade (Figure 2G). We concluded that huCD39 mAb effectively enhanced T-cell function by blocking the degradation of ATP.

### The huCD39 mAb exhibited effective anti-tumor efficacy against xenografts as a monotherapy and in combination with the PD-L1 antibody

**Figure 3:**
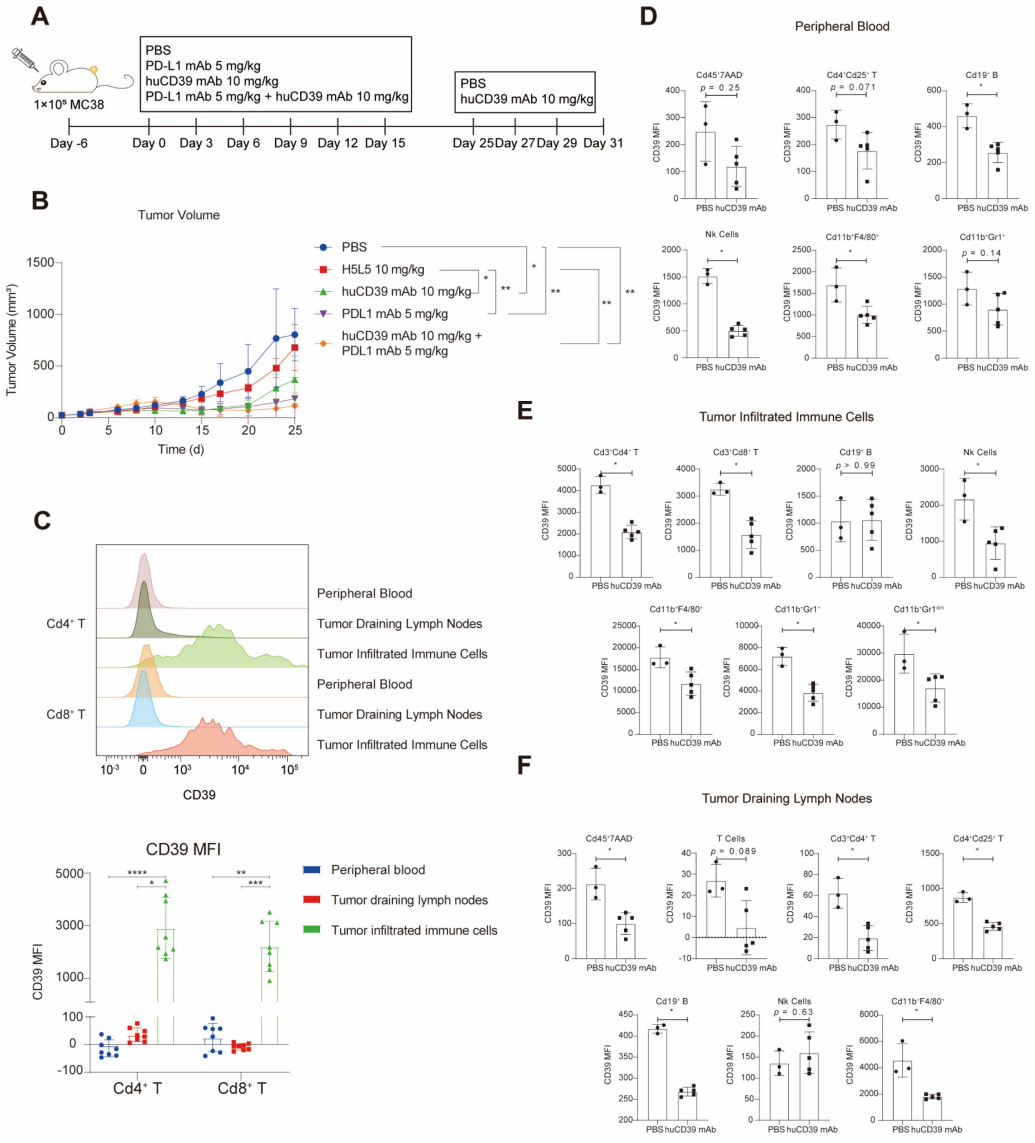
** HuCD39 mAb showed effective anti-tumor efficacy against xenografts in human CD39 knock-in mice.** (A-B) Mice (n = 5) were injected subcutaneously with MC38 (1E5) cells, 6 days after inoculation. Mice were treated with H5L5 (another CD39 antibody, 10 mg/kg), huCD39 mAb (10 mg/kg), PD-L1 mAb (5 mg/kg) or combination of huCD39 mAb with PD-L1 mAb. Tumor growth was monitored every 2-3 days. Data are shown as mean ± SD. *, P < 0.05; **, P < 0.01 by two-way ANOVA test, followed by Tukey's multiple comparisons test. Additive three doses of huCD39 mAb were given on Day 25, 27 and 29, and tumor samples were harvested on Day 31 for flow cytometry. (C) CD39 expression on Cd4^+^ T and Cd8^+^ T collected from peripheral blood, tumor draining lymph nodes and tumor infiltrated immune cells were compared via flow cytometry. Data are shown as mean ± SD. *, P < 0.05; **, P < 0.01; ***, P < 0.001; ****, P < 0.0001 by one-way ANOVA test, followed by Dunn's multiple comparisons test. (D-F) CD39 expression was compared in different types of immune cells after huCD39 mAb treatment from peripheral blood (D), tumor infiltrated immune cells (E) and tumor draining lymph nodes (F). Data are shown as mean ± SD. Two mice from PBS group were euthanized on Day 25 and Day 29 respectively due to excessive tumor volume. *, P < 0.05 by Mann-Whitney test.

To assess the efficacy of the huCD39 mAb *in vivo*, we evaluated the anti-tumor efficacy of targeting CD39 in human *CD39* knock-in (KI) mice (purchased from Biocytogen) subcutaneously injected with MC38 tumor cells. PBS vehicle or different antibodies were administered intraperitoneally (i. p.) every 3 days for six doses (Figure 3A). Treatment with the huCD39 mAb antibody produced a significant reduction in tumor growth that was superior to another anti-CD39 antibody (H5L5). Additionally, the combination of huCD39 mAb with an anti-PD-L1 antibody showed slight improvement in anti-tumor efficacy compared to single treatments (Figure 3B). Mice treated with anti-PD-L1 alone showed a little decrease in body weight, but mice treated with anti-CD39 antibodies or combination of both antibodies were not affected (Figure S6G). To further understand the mechanism underlying huCD39 mAb mediated anti-tumor activity, another three doses of huCD39 mAb were administered before euthanasia. Minimal alterations in immune cell recruitment were observed in the TME, peripheral blood (PB) or tumor-draining lymph nodes (dLNs) (Figures S6A-C). Further analysis revealed that CD39 expression was higher in tumor-infiltrating T cells than in T cells from PB or dLN (Figure 3C). Decreased expression of the CD39 protein was also detected in tumor infiltrating T cells, macrophages, NK cells, and MSDCs upon huCD39 mAb treatment, which was similar in PB and dLN (Figures 3D-F). Therefore, the anti-CD39 antibody modulated the entire TME to attenuate CD39-mediated immunosuppression. Expression of CD25 was not affected in T cells from TME, PB or dLN (Figures S6D-F).

### Bioinformatic analysis revealed *CD39* as a potential target for CAR-T therapy in ovarian cancer

**Figure 4:**
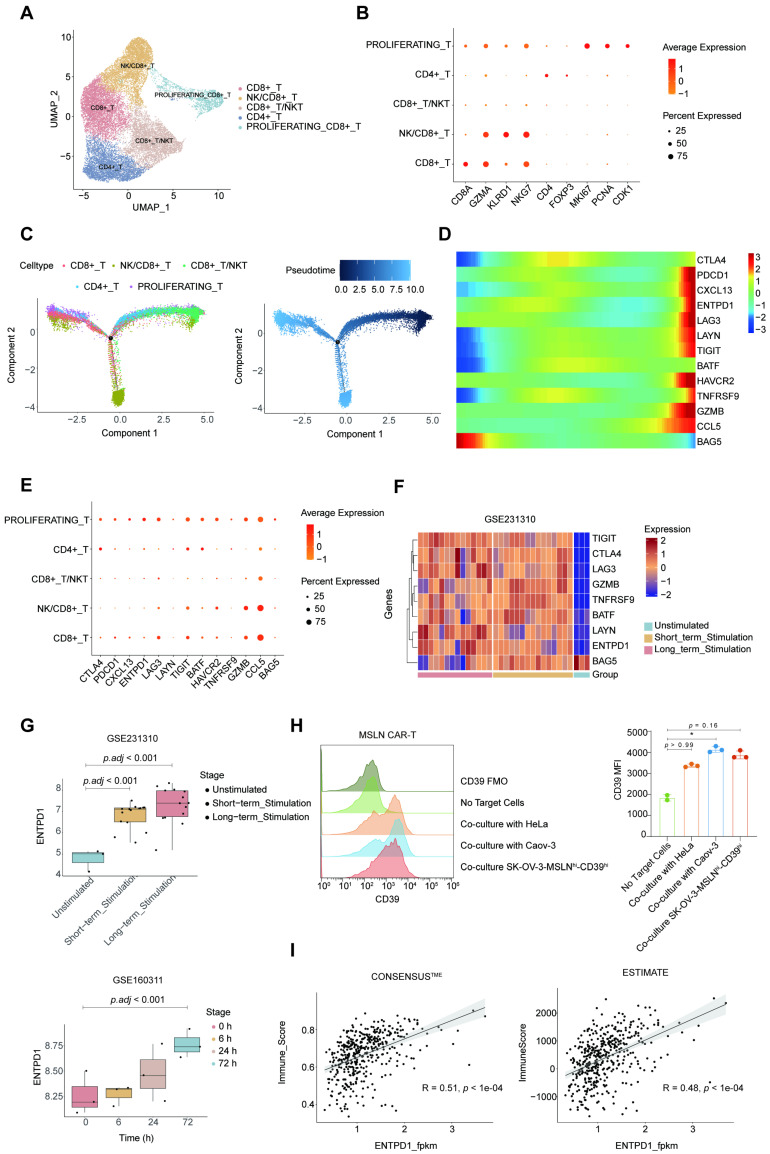
** Bioinformatic analysis identified elevated CD39 on tumor-specific T cells and activated CAR-T cells.** (A) Umap of T cells in GSE184880 dataset, colored by T cell subpopulations. (B) Dot plot showing markers in T cell subpopulations. (C) Pseudotime trajectory analysis of T cell subpopulations. (D) Heat map of differential genes between tumor-specific and bystander CD8^+^ T along pseudotime. (E) Dot plot showing differential genes between tumor-specific and bystander CD8^+^ T in T cell subpopulations. (F) Heat map of differential genes between tumor-specific and bystander CD8^+^ T cells which are also significant in GSE231310 dataset. (G) Boxplot showing *ENTPD1* (*CD39*) expression on CAR-T cells after co-culture with tumor cells in GSE231310 (top) and GSE160311 (bottom) datasets. (H) Expression of CD39 on MSLN CAR-T after co-culture with MSLN positive cancer cell lines via flow cytometry. Data are shown as mean ± SD. *, P < 0.05 by one-way ANOVA test, followed by Dunn's multiple comparisons test. Experiment was repeated on over three different donors. Histogram showing 2-3 technical repeats in one representative donor. (I) Correlation between *ENTPD1* (*CD39*) expression and immune scores calculated by Consensus^TME^ (left) and ESTIMATE (right) from TCGA ovarian cancer cohort.

To provide a rationale for combining huCD39 mAb with CAR-T therapy in ovarian cancer, we analyzed the publicly available dataset GSE184880 (single-cell transcriptome data on HGSOC and matched non-malignant ovarian tissue). We identified five main clusters referred to as B cells, epithelial cells, myeloid cells, stromal cells, and T cells (Figures S7A-B). ENTPD1 (CD39) was widely expressed in ovarian tissue, and was elevated in cancer tissue compared to normal tissue (Figure S7C). We re-clustered T cells into five subgroups. Figures 4A-B show the identification of subpopulations, including a proliferating subtype (marked by *MKI67*, *PCNA,* and *CDK1*). The subpopulation proportions of T cells in different patients are shown in Figure S7D. We computed a pseudotime trajectory of the T-cell cluster in cancer tissue, with the proliferating CD8^+^ T subgroup located at one of the endpoints along the trajectory (Figure 4C). We then plotted differential genes between tumor-specific and bystander CD8^+^ T cells according to Liu *et al.* (Figure 4D) 48. *CXCL13*, which is a well-recognized tumor-specific T-cell marker was elevated in proliferating CD8^+^ T cells. Other exhaustion related markers such as *ENTPD1 (CD39)*, *PDCD1*, *LAG3,* and *TIGIT* were also expressed at relatively high levels in this cluster (Figure 4E and Figure S7E).

Because CAR-T cells recognize tumor-associated antigens on tumor cells, we wondered whether similar changes would occur in CAR-T cells when they entered the TME as tumor-specific T cells. Two publicly available datasets (GSE231310 and GSE160311) revealed the elevated CD39 levels in CD19 CAR-T cells cocultured with the CD19^+^ acute lymphocytic leukemia cell line Nalm6 and in anti-EGFR CAR-T cells cocultured with EGFR^+^ A549 cells (Figures 4F-G and Figure S7F). We used flow cytometry to test CD39 expression on antigen-challenged MSLN CAR-T cells, and the results revealed that MSLN CAR-T cells cocultured with MSLN-positive tumor cells also upregulated CD39 expression (Figure 4H Figures S8A and S8C). We further detected a positive correlation between *ENTPD1 (CD39)* expression and immune infiltration scores using the TCGA-OV data, and the results indicated that infiltration of immune cells may lead to the upregulation of ENTPD1 (CD39) in the TME (Figure 4I).

Taken together, these results suggest that CD39 is abundant in ovarian cancer, but it may also serve as an obstacle to CAR-T-cell function. Targeting CD39 likely enhances the effect of current immunotherapies (i.e. immune checkpoint blockade and CAR-T-cell therapy) in ovarian cancer.

### HuCD39 mAb enhanced the anti-tumor activity of CAR-T in xenografts

**Figure 5:**
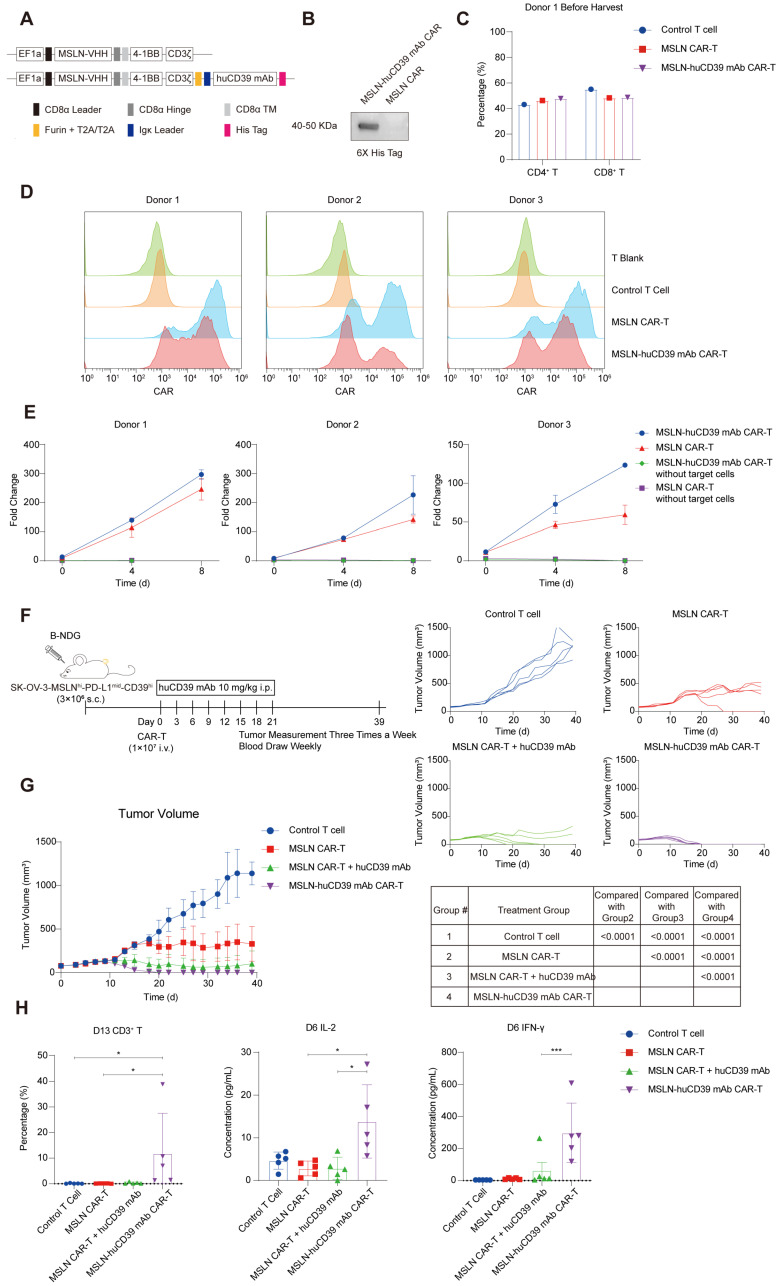
** Combination of huCD39 mAb with MSLN CAR-T therapy improved anti-tumor efficacy in ovarian cancer.** (A) Schematic representation of MSLN CAR and huCD39 mAb antibody-secreting CAR constructs. MSLN CAR (top) represents CAR targeting MSLN, MSLN-huCD39 mAb CAR (bottom) represents MSLN CAR-T secreting huCD39 mAb. (B) Supernatants from MSLN CAR-T and MSLN-huCD39 mAb CAR-T of donor 1 were collected, antibodies were detected via western blotting. (C) CD4/CD8 proportion in CAR-T cells from donor 1 was detected via flow cytometry before harvest for freezing. (D) T cells were incubated with 5 mg/kg MSLN-his protein and APC-conjugated anti-his secondary antibody, CAR expression was measured via flow cytometry from three different donors. (E) After three rounds of coculturing with SK-OV-3-MSLN^hi^-CD39^hi^ ovarian cells, CAR^+^ T cells were numbered based on cell counting using AO/PI staining combined with flow cytometry, cumulative fold change was calculated. CAR-T without targeted cells were used as negative controls. Data are shown as mean ± SD. (n = 1-3 technical replicates). (F) Schematic for subcutaneously inoculation of 3E6 SK-OV-3-MSLN^hi^-CD39^hi^ ovarian cells in B-NDG mice and treatment. (G) Mice (n = 5) were injected subcutaneously with SK-OV-3-MSLN^hi^-CD39^hi^ ovarian cells, and treated with 1E7 MSLN CAR-T cells when tumor volume reached 75-80 mm^3^. Systematically administration of antibodies started on the same day of CAR-T infusion. Totally 8 doses of antibody were given intraperitoneally. Tumor growth was monitored every three days. Data are shown as mean ± SD. ****, P < 0.0001 by two-way ANOVA test, followed by Tukey's multiple comparisons test. (H) Weekly blood test showing percentage of human CD3^+^ T in B-NDG mice on Day 13 after CAR-T infusion and IL-2 and IFN-γ level on Day 6 after CAR-T infusion. Data are shown as mean ± SD. *, P < 0.05; ***, P < 0.001 by one-way ANOVA test, followed by Dunn's multiple comparisons test.

Building on the observation of elevated CD39 expression in ovarian cancer and the elevated CD39 expression in activated CAR-T cells, we constructed huCD39 mAb secreting MSLN CAR-T cell (Figure 5A and Figure 5D). The constructed CAR lentiviruses were used to infect T cells from different donors, and secreted antibodies with His-tags were collected from the supernatants of CAR-T cells from donors 1 and 6 in the process of large-scale culture. After purification using an NI-NTA-Agarose column, the purified antibodies were detected via western blotting (Figures 5B). *In vitro* experiments revealed robust expansion of antibody-secreting CAR-T cells after three rounds of coculture with SK-OV-3-MSLN^hi^-CD39^hi^ ovarian cancer cells compared with ordinary CAR-T cells (Figure 5E and Figures S8B-C). However, the secretion of antibodies did not alter the cytolytic activity or memory phenotype of CAR-T cells (Figures S9A-B).

To assess whether antibodies improved CAR-T-cell function *in vivo*, the efficacy of CAR-T-cell therapy combined with the systematic administration of antibodies or antibody-secreting CAR-T cells was compared. There was not much difference in CD4/CD8 ratio in CAR-T cells from donor 1 (Figure 5C). We inoculated B-NDG mice with SK-OV-3-MSLN^hi^-CD39^hi^ ovarian cancer cells subcutaneously. 1E7 CAR-T cells were used in tumor bearing mice when the tumor volume reached an average of 75-80 mm^3^, and antibodies were administered intraperitoneally every three days (Figure 5F). The second-generation MSLN CAR-T-cell secreting anti-CD39 mAb effectively eradicated tumors on days 18-20 after infusion, when MSLN CAR-T cells alone were less vigorous. Additionally, the secreted forms of antibodies were much more efficient than systemic infusion as shown in our results (Figure 5G). Body weight in Control T cell group and MSLN CAR-T cell group were decreased (Figure S9C).

We also evaluated the histomorphology of vital organs, and no evident damages were observed (Figure S9D). Periodic blood tests revealed that the highest proportion of CD3^+^ T cells was presented in the second week after infusion, whereas the level of IL-2 and IFN-γ peaked in the first week (Figures S9E-F). The increased levels of IL-2 and IFN-γ as well as the CD3^+^ T cell proportion in the antibody-secreting CAR-T-cell group suggested that the secreted forms of antibodies increased the immune response of MSLN CAR-T cells against ovarian tumor xenografts (Figure 5H).

**Figure 6:**
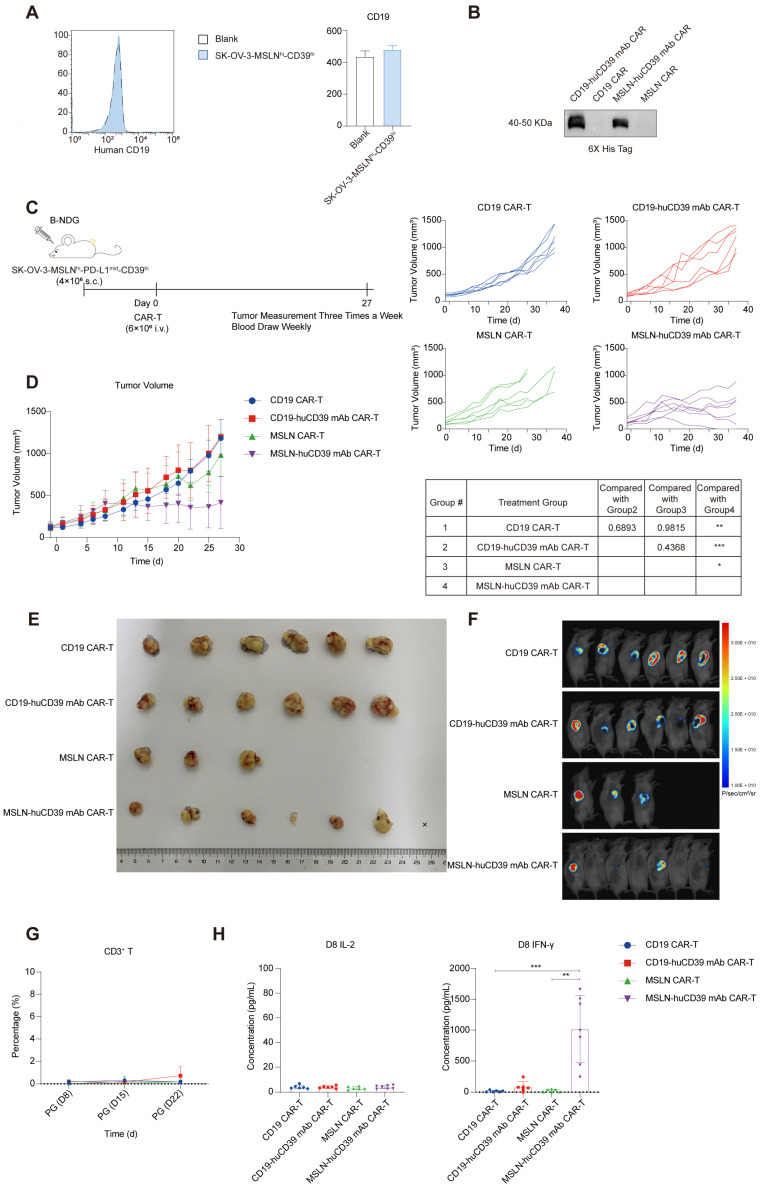
** Lower dose of huCD39 mAb secreting MSLN CAR-T inhibited ovarian cancer growth.** (A) CD19 expression in SK-OV-3-MSLN^hi^-CD39^hi^ ovarian cells was measured via flow cytometry. (B) Supernatants from CD19 CAR-T, CD19-huCD39 mAb CAR-T, MSLN CAR-T and MSLN-huCD39 mAb CAR-T of donor 6 was collected, antibodies were detected via western blotting. (C) Schematic for subcutaneously inoculation of 4E6 SK-OV-3-MSLN^hi^-CD39^hi^ ovarian cells in B-NDG mice and treatment. (D) Mice were injected subcutaneously with SK-OV-3-MSLN^hi^-CD39^hi^ ovarian cells, and treated with 6E6 CAR-T cells when tumor volume reached 115-135 mm^3^. Tumor growth was monitored every three days. Data are shown as mean ± SD. ****, P < 0.0001 by two-way ANOVA test, followed by Tukey's multiple comparisons test. (E) Images of tumor tissue after treatment with CD19 CAR-T (n = 6), CD19-huCD39 mAb CAR-T (n = 6), MSLN CAR-T (n = 6), MSLN-huCD39 mAb CAR-T (n = 7). Three mice in MSLN CAR-T were euthanized on day 20 after CAR-T-cell infusion because the tumors had broken poorly and the wounds had not healed. (F) Bioluminescence imaging of tumors on day 27 after treatments. (G) Weekly blood test showing changes of CD3^+^ T proportion via flow cytometry. (H) Weekly blood test showing IL-2 and IFN-γ levels on Day 8 after CAR-T infusion. Data are shown as mean ± SD. **, P < 0.01; ***, P < 0.001 by one-way ANOVA test, followed by Dunn's multiple comparisons test.

To further assess the anti-tumor efficacy of huCD39 mAb-mediated CAR-T-cell therapy, we inoculated B-NDG mice with 4E6 SK-OV-3-MSLN^hi^-CD39^hi^ ovarian cells subcutaneously. Flow cytometry revealed that SK-OV-3-MSLN^hi^-CD39^hi^ does not express CD19 (Figure 6A). We constructed CAR-T targeting CD19 as an irrelevant CAR-T cell (Figure S10A). CD19 CAR-T has no specific killing of SK-OV-3-MSLN^hi^-CD39^hi^ tumor cells, and CD19 CAR-T does not expand after coculturing with tumor cells (Figures S10B-C). Before harvest, we found that huCD39 mAb-secreting CD19 CAR-T had more CD4^+^ T and less CD8^+^ T, but this is not observed in huCD39 mAb-secreting MSLN mAb CAR-T (Figure S10D). CD19 and MSLN CAR-T cells alone or CAR-T cells were produced from another donor (Donor 6). Secretion of huCD39 mAb in supernatants of huCD39 mAb-secreting CD19 CAR-T and huCD39 mAb-secreting MSLN CAR-T cells was confirmed via western blotting (Figure 6B). One dose of 6E6 CAR-T cells was infused when the tumor volume reached a relatively large size (average of 115-135 mm^3^) (Figure 6C). HuCD39 mAb-secreting MSLN CAR-T cells effectively inhibited tumor growth after infusion when MSLN CAR-T-cell therapy alone or CD19 CAR-T-cell therapy did not inhibit tumor growth (Figures 6D-E). Bioluminescence imaging also indicated that the huCD39 mAb-secreting MSLN CAR-T-cell had the most efficient tumor inhibition (Figure 6F). HuCD39 mAb-secreting MSLN CAR-T cells exhibited significantly higher levels of IFN-γ than other groups, but CD3^+^ T cells or IL-2 was not detected in any group (Figures 6G-H and Figure S10F). Body weight was relatively higher in huCD39 mAb-secreting MSLN CAR-T group, probably due to the efficient inhibition of tumor growth (Figure S10E). We additionally evaluated liver function, and the results revealed normal liver function after treatment (Figure S10G). We also investigated organ histomorphology after CAR-T-cell therapy and found no obvious damage to vital organs (Figure S10H).

## Discussion

In the current study, a novel anti-CD39 nanobody huCD39 mAb was developed. This antibody with potent blocking ability successfully alleviated CD39 mediated immunosuppression and activated the anti-tumor response in our *in vitro* and *in vivo* models. Elevated CD39 expression was detected in ovarian cancer tissue and in activated CAR-T cells. The combination of CAR-T-cell therapy with systematic administration of a CD39 antibody yielded promising anti-tumor efficacy against ovarian cancer, and even greater efficacy was observed when the antibody was administered in a secreted form. Our findings demonstrate the ability of a novel anti-CD39 antibody to enhance the efficacy of CAR-T cells in ovarian cancer and provide a valuable strategy for optimizing the behavior of CAR-T cells in solid tumors.

CD39 is a promising target in tumor immunotherapy. Multiple studies have confirmed the efficacy of anti-CD39 antibodies in tumor xenograft models 12,15,16. However, these full-length antibodies are not suitable for CAR construction due to their excessively long sequences. A recent study developed an anti-CD39 nanobody, but it did not evaluate the *in vivo* efficacy of this nanobody 19. Our huCD39 mAb shares advantages with nanobodies, especially its small molecular size, stability, excellent dissolubility, strong penetration and high affinity 49. Notably, we further validated the function of huCD39 mAb in promoting T-cell proliferation and tested its efficacy in mouse models. Our huCD39 mAb was superior to the currently available full-length antibodies in terms of its blocking ability and anti-tumor function.

In terms of the antitumor mechanism of CD39 antibodies, it has been indicated that CD39 blockade on macrophages and monocytes led to the activation of the NALP3 inflammasome and the release of IL-18 and IL-1β to exert anti-tumor effects 15. We performed an *in vivo* experiment using a classical MC38 mouse model, and the results suggested that the novel huCD39 mAb modulated the entire TME to switch into an immune responsive state by downregulating CD39 in immune cells. This finding is consistent with another study in which the CD39 inhibitor POM-1 induced only minor changes in immune cell components but may affects the entire TME by converting it into a “hot” tumor 50. Immunofluorescence analysis indicated that huCD39 mAb induced internalization. This process likely led to the degradation of the CD39 antigen, but further exploration is needed.

The study of tumor-infiltrating lymphocytes has highlighted a distinct subpopulation of CD39^+^ T cells that undergo clonal expansion upon antigen recognition 11,51. Compared to their CD39^-^ counterparts, CD39^+^ conventional T (Tconv) cells exhibit enhanced cytotoxic effects and release increased levels of proinflammatory cytokines 52. The study by Zou *et al*. also revealed that CD39^+^ HBV-specific CAR-T cells were more potent in controlling tumors than CD39^-^ CAR-T cells 53. Collectively, these results suggest that elevated CD39 may serve as a marker of the immune response in tumor immunology. Given that CAR-T cells are also antigen specific, it can be postulated that CAR-T cells may resemble antigen-specific CD8^+^ T cells when they enter the tumor environment. Notably, the current study observed an increase in CD39 expression on CAR-T cells after they were cocultured with target cells. Given its role in immunosuppression, the high level of CD39 on antigen responsive T cells and CAR-T cells makes it a potential target to enhance the efficacy of CAR-T therapy 54.

CAR-T therapy has made some progress in solid tumors, but showed rather unpleasant outcomes in ovarian cancer patients 21,55. The immunosuppressive tumor environment is one of the main challenges of CAR-T therapy in ovarian cancer, and elevated expression of purinergic pathway molecules in cancer further exacerbates this problem. CAR-T cells engineered to express adenosine deaminase, an enzyme which further catalyzed adenosine into inosine, exhibited enhanced potency 56. Several studies also combined adoptive cell therapy with CD39 blockade. Potenza *et al*. revealed that knockout of *CD39* in T cells targeting HER-2 enhanced the anti-tumor activity in colorectal tumoroid 57. CD39 inhibitor-pretreated CAR-T cells achieved improved anti-tumor efficacy 58. However, genome editing of CAR-T cells leads to impaired viability and an unmeasurable off-target risk. The application of small molecule inhibitors of CD39 is limited by their short half-life and low specificity. Although improvements in these strategies have been reported, our strategy provides patients with alternative options. We performed two independent animal experiments, differing in dosage and the source of T cells. Both of them confirmed the efficacy of huCD39 mAb secreting CAR-T cells in B-NDG mouse models inoculated with ovarian cancer cells.

In the current study, we employed a novel strategy that combines CAR-T cells with anti-CD39 antibodies, achieving significant tumor inhibition in ovarian tumor xenografts. We further observed a more potent anti-tumor effect from the secreted form of the antibody compared to systematically administration. We presume that anti-CD39 antibody-secreting CAR-T cells can take full advantage of the ability of CAR-T cells to target tumors, and this antibody release pattern may contribute to a more uniform distribution of antibodies in the TME. Drawing from the findings of Rafiq, S. *et al*., the superior efficacy of antibody-secreting CAR-T cells was due to locally enriched antibodies in the TME which further reflects the feasibility and necessity of our combination strategy 59. Moreover, this localized antibody secretion reduces its “off-target” effect in the periphery and decreases the production of anti-drug antibodies, thereby delaying the onset of drug resistance, which indicates the safety of this strategy. Despite its effectiveness in treating ovarian cancer in current settings, more models, such as organoid and PDX model, are needed to validate the effectiveness of the huCD39 mAb and the huCD39 mAb secreting CAR-T cells. Future research is also needed to clarify the indications for CD39 antibody-secreting CAR-T therapy in clinical practice.

## Supplementary Material

Supplementary figures and tables.

## Abbreviations

CAR-T: chimeric antigen receptor T
TME: tumor microenvironment
MSLN: mesothelin
ECD: extracellular ligand recognition domain
BLI: Biolayer interferometry
ELISA: enzyme-linked immunosorbent assay
PBMC: peripheral blood mononuclear cell
HGSOC: high-grade serous ovarian cancer
i.v.: intravenously
i.p.: intraperitoneally
ADCC: antibody-dependent cellular cytotoxicity
CDC: complement-dependent cytotoxicity
KI: knock-in
PB: peripheral blood
dLN: draining lymph nodes
