# Supplementary material for: Development of a CD39 nanobody and its enhancement to chimeric antigen receptor T cells efficacy against ovarian cancer in preclinical studies
Source: Theranostics. 2024 Sep 30;14(16):6249–67. doi: 10.7150/thno.97590 (PMC11488103; doi:10.7150/thno.97590)
Supplement: Supplementary file 1 — Supplementary figures and tables. [file thnov14p6249s1.pdf]

Supplementary Materials

Supplementary Figures:

Figure S1. Screening nanobodies against human CD39.

Figure S2. Twenty-four selected antibodies were tested on their specific binding to CD39 on cell membrane via flow cytometry.

Figure S3. HuCD39 mAb and H5L5 induced internalization.

Figure S4. Characterization of humanized CD39 antibody (huCD39 mAb) targeting CD39.

Figure S5. HuCD39 mAb reversed CD39 induced T cell dysfunction.

Figure S6. Alteration of immune cell composition after huCD39 mAb treatment in MC38 mouse model.

Figure S7. Bioinformatic analysis identified elevated CD39 in ovarian cancer.

Figure S8. Expression of MSLN and CD39 in tumor cell lines.

Figure S9. *In vitro* and *in vivo* function of huCD39 mAb secreting MSLN CAR-T.

Figure S10. *In vitro* and *in vivo* function of huCD39 mAb secreting CAR-T targeting CD19 and MSLN.

Supplementary Tables:

Table S1. EC50 of 24 tested anti-CD39 antibodies against CD39 protein via ELISA.

Table S2. Affinity of 24 tested anti-CD39 antibodies against CD39 protein via BLI.

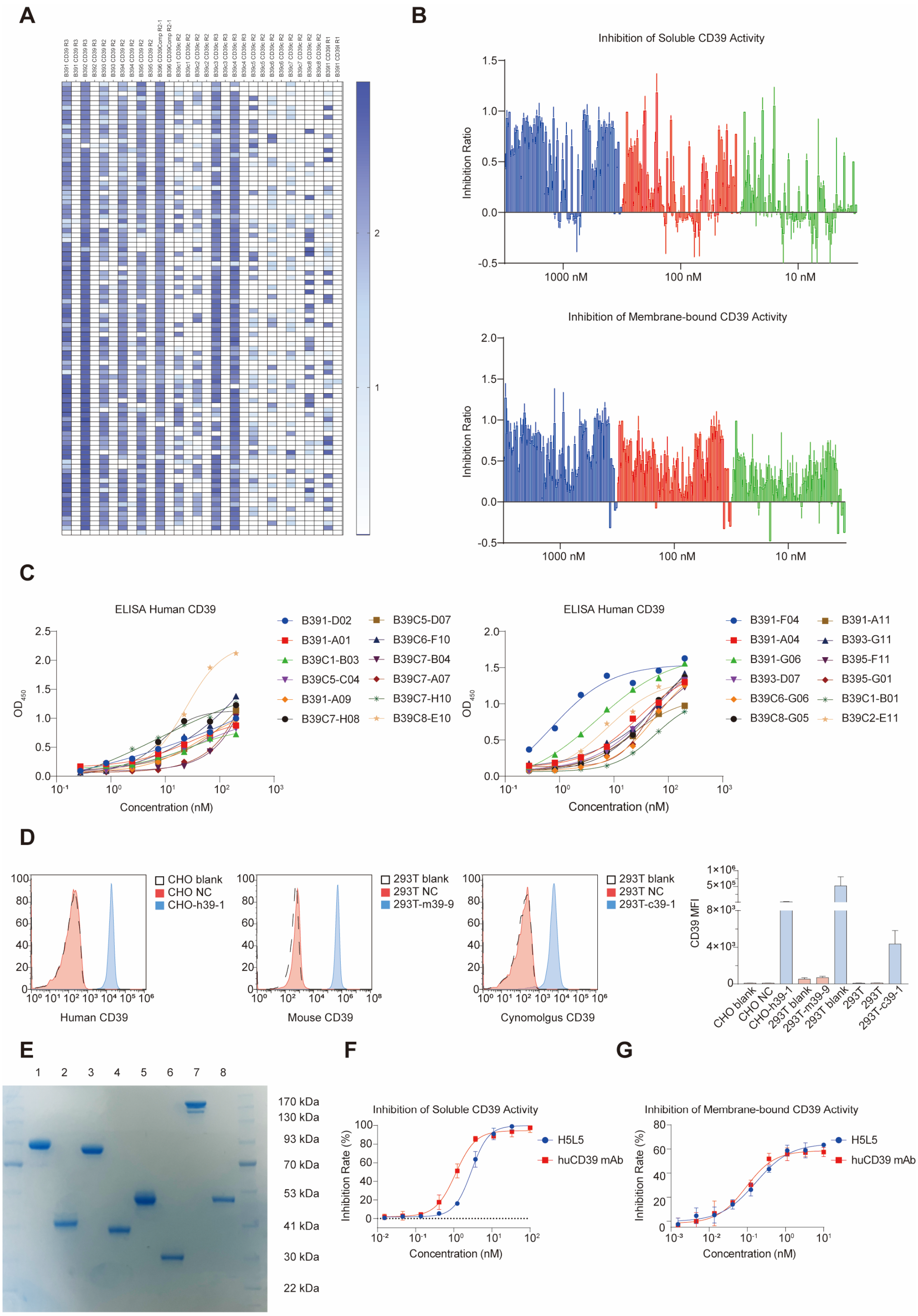

Figure S1. Screening of nanobodies against human CD39.

(A) ELISA revealed that over 400 phage-infected could bind to CD39-his protein (left column) but not IgG-FC protein (right column). (B) Blockade of enzymatic activity on soluble and membrane-bound CD39 were tested on 87 unique antibodies. (C) Binding to CD39-his protein of 24 selected antibodies were tested via ELISA. (D) Human *CD39* was overexpressed on CHO cell lines, mouse *CD39* and

cynomolgus *CD39* were overexpressed on 293T cells, expression was tested via flow cytometry. Data are shown as mean ± SD. (E) SDS-PAGE results of purified proteins under non-reducing (NR) and reducing (R) conditions. Lane 1, CD39 mAb (NR); Lane 2: CD39 mAb (R); Lane 3: huCD39 mAb (NR); Lane 4: huCD39 mAb (R); Lane 5: IgG-FC (NR); Lane6: IgG-FC (R); Lane 7: H5L5 (NR); Lane 8: H5L5 (R). (F-G) Soluble (F) or membrane-bound CD39 (G) were blocked with huCD39 mAb or H5L5 indicated concentrations of antibodies. Data are shown as mean ± SD (n = 3).

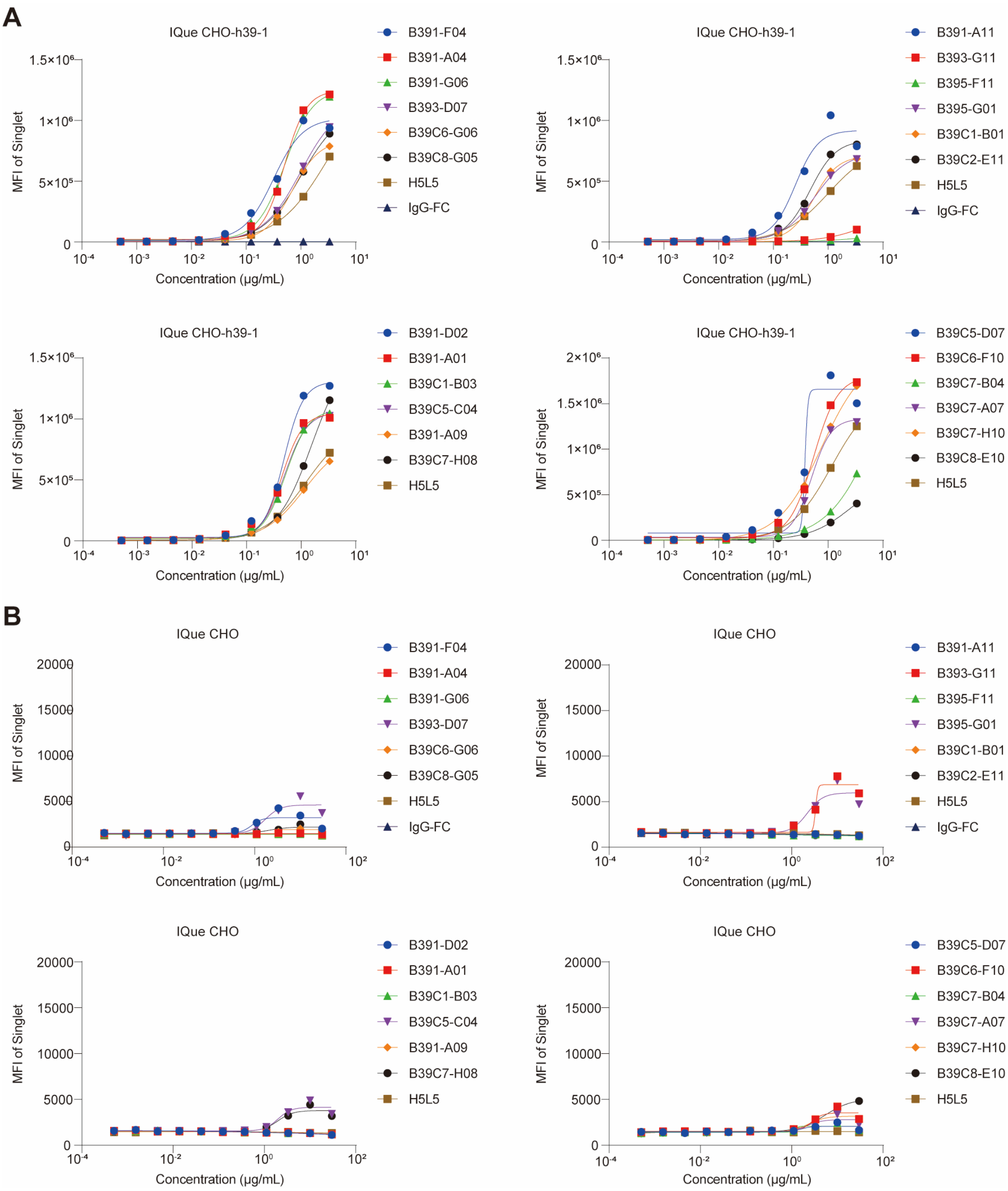

Figure S2. Twenty-four selected antibodies were tested on their specific binding to CD39 on cell membrane via flow cytometry.

(A) Antibodies were able to bind human CD39 in CHO-h39-1 cells via IQue-based flow cytometry. (B) Non-specific binding was not detected in CHO cell lines via IQue-based flow cytometry.

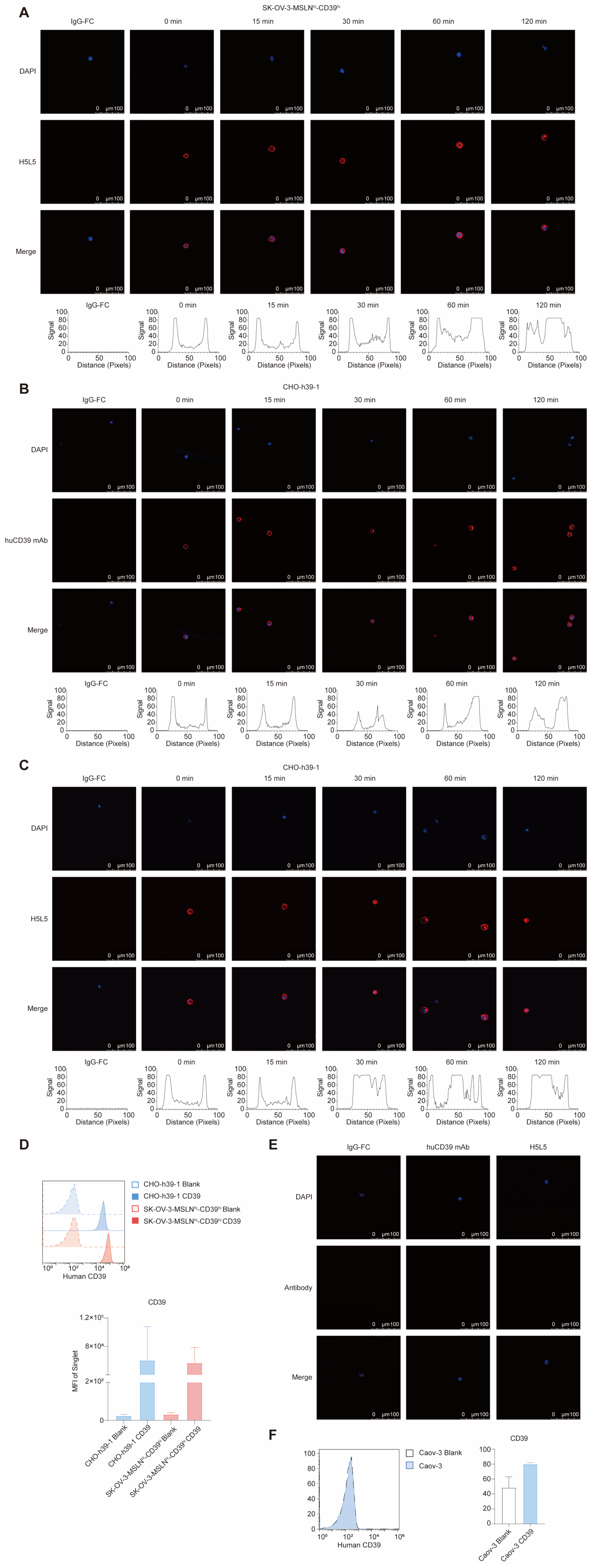
A
SK-OV-3-MSLNhi-CD39hi
IgG-FC
0 min
15 min
30 min
60 min
120 min
DAPI
H5L5
Merge
Signal
Distance (Pixels)
B
CHO-h39-1
huCD39 mAb
C
CHO-h39-1
D
CHO-h39-1 Blank
CHO-h39-1 CD39
SK-OV-3-MSLNhi-CD39hi Blank
SK-OV-3-MSLNhi-CD39hi CD39
Human CD39
CD39
MFI of Singlet
E
IgG-FC
huCD39 mAb
H5L5
DAPI
Antibody
Merge
F
Caov-3 Blank
Caov-3
Human CD39
CD39
Caov-3 Blank
Caov-3 CD39

Figure S3. HuCD39 mAb and H5L5 induced internalization.

(A) Internalization of H5L5 in SK-OV-3-MSLN^{hi}-CD39^{hi} was detected via immunofluorescence staining. (B-C) Internalization of huCD39 mAb (B) and H5L5 (C) in CHO-h39-1 was detected via immunofluorescence staining. (D) Expression of human CD39 in SK-OV-3-MSLN^{hi}-CD39^{hi} and CHO-h39-1 was compared via flow cytometry. (E) Immunofluorescence staining of CD39 via huCD39 mAb and H5L5 was performed in Caov-3 cells. (F) Expression of human CD39 in Caov-3 was measured via flow cytometry.

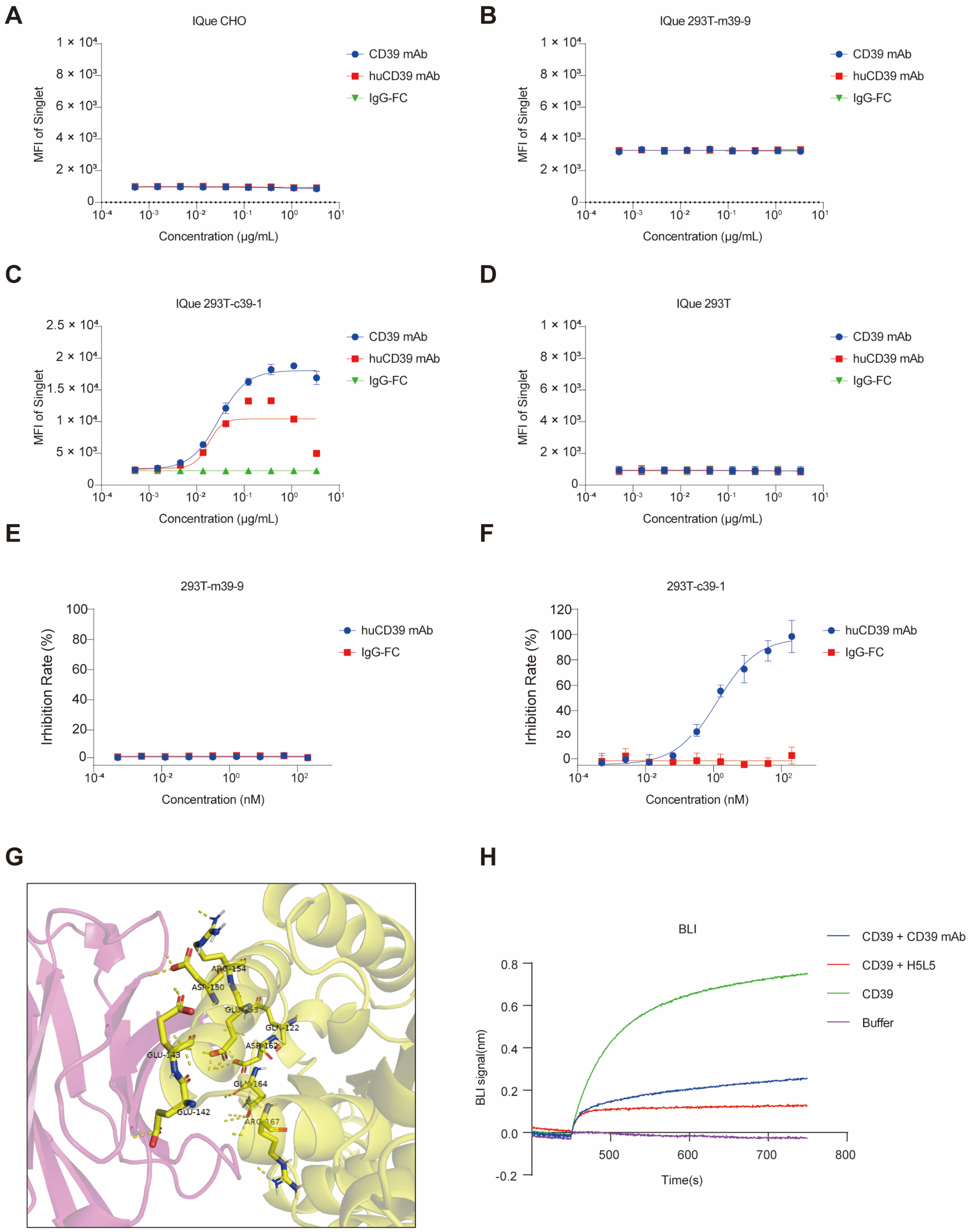

Figure S4. Characterization of humanized CD39 antibody (huCD39 mAb) targeting CD39.

(A) Non-specific binding was not detected on CHO cell lines via IQue-based flow cytometry. (B-D) Binding of huCD39 mAb to mouse (B) and cynomolgus (C) CD39 on cell membrane was measured via flow cytometry, and non-specific binding was not detected on 293T cells (D). (E-F) Blocking of membrane-bound mouse and cynomolgus CD39 enzymatic activity was determined. (G) Predicted interaction sites of CD39 (yellow) and huCD39 mAb (pink). (H) Epitope competition of CD39 mAb and H5L5 via BLI. Data are shown as mean ± SD (n = 3).

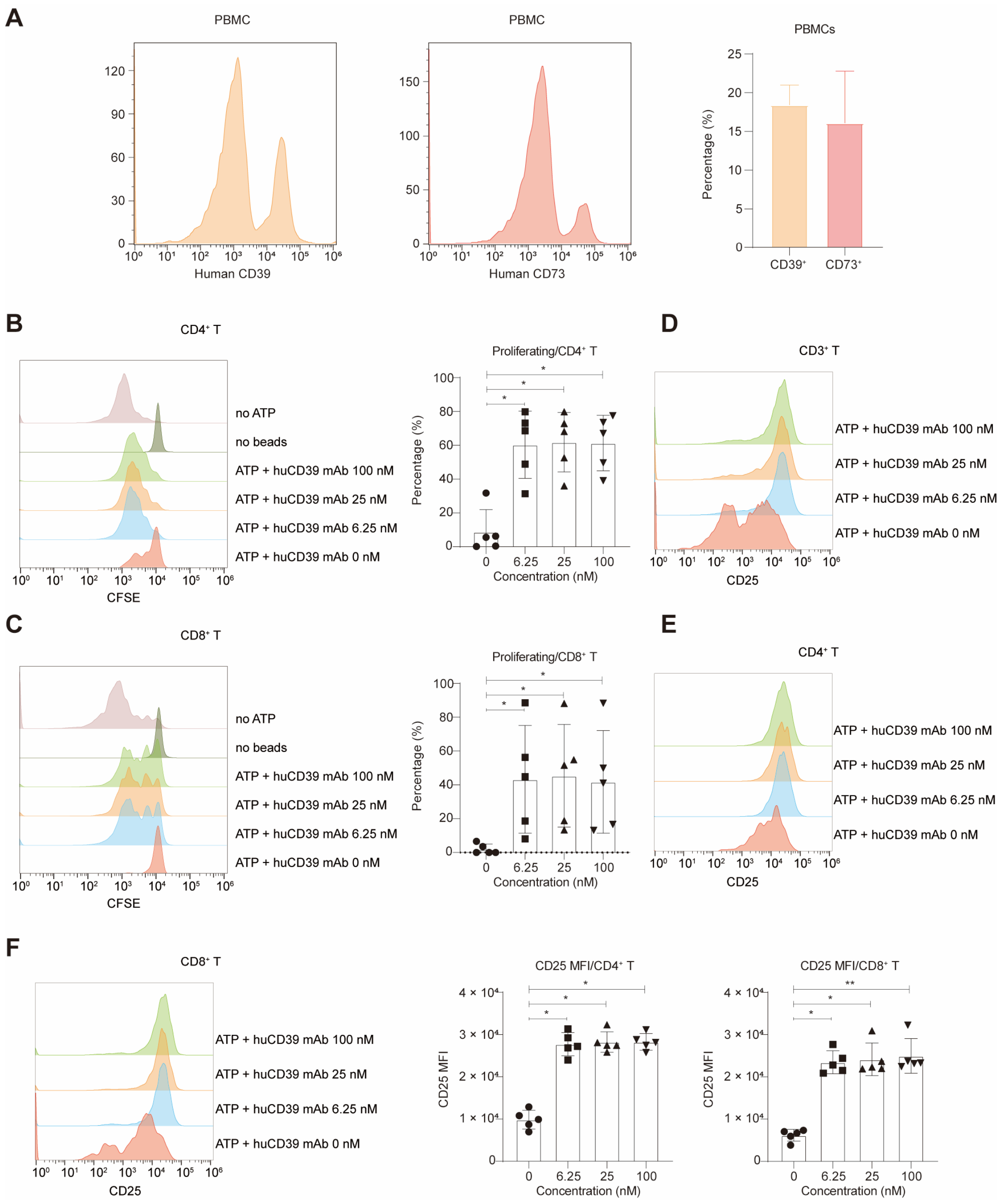

Figure S5. HuCD39 mAb reversed CD39 induced T cell dysfunction.

(A) Expression of CD39 and CD73 in PBMCs was detected via flow cytometry. (B-C) Proliferation was measured via CFSE dilution assay through flow cytometry on CD4$^+$ T (B) and CD8$^+$ T (C) respectively. (D-F) CD25 MFI was assessed via flow cytometry on CD3$^+$ T (D), CD4$^+$ T (E) and CD8$^+$ T (F) respectively. Data are shown as mean ± SD. *, $P < 0{\cdot}05$; **, $P < 0{\cdot}01$ by one-way ANOVA test, followed by Dunn's multiple comparisons test. The data presented is representative of samples collected from five distinct donors.

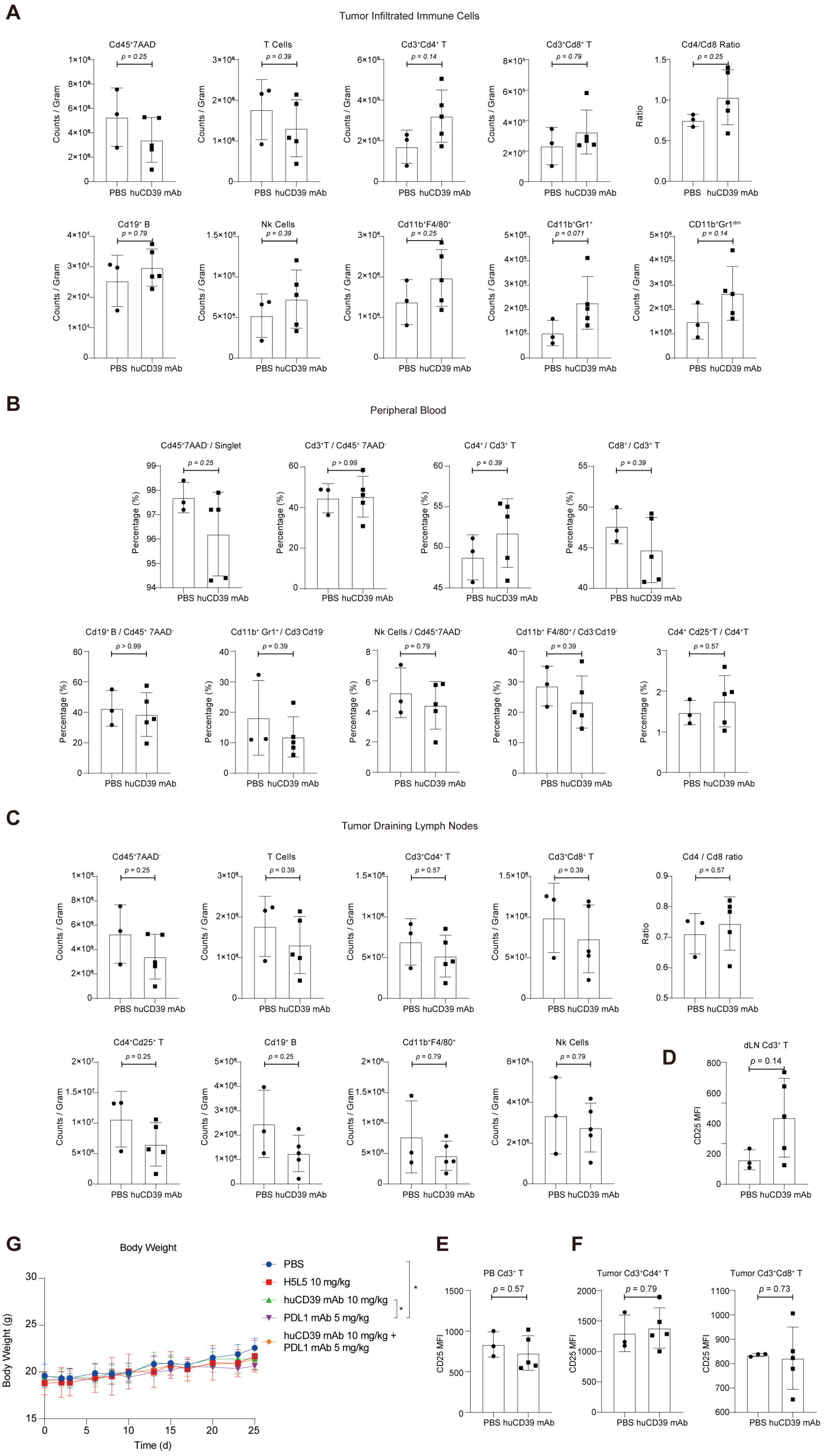
A
Tumor Infiltrated Immune Cells
Cd45+7AAD-
p = 0.25
T Cells
p = 0.39
Cd3+Cd4+ T
p = 0.14
Cd3+Cd8+ T
p = 0.79
Cd4/Cd8 Ratio
p = 0.25
Cd19+ B
p = 0.79
Nk Cells
p = 0.39
Cd11b+F4/80+
p = 0.25
Cd11b+Gr1+
p = 0.071
CD11b+Gr1dim
p = 0.14
Counts / Gram
Ratio
PBS huCD39 mAb
B
Peripheral Blood
Cd45+7AAD- / Singlet
p = 0.25
Cd3+T / Cd45+ 7AAD-
p > 0.99
Cd4+ / Cd3+ T
p = 0.39
Cd8+ / Cd3+ T
p = 0.39
Cd19+ B / Cd45+ 7AAD-
p > 0.99
Cd11b+ Gr1+ / Cd3-Cd19-
p = 0.39
Nk Cells / Cd45+7AAD-
p = 0.79
Cd11b+ F4/80+ / Cd3-Cd19-
p = 0.39
Cd4+ Cd25+T / Cd4+T
p = 0.57
Percentage (%)
PBS huCD39 mAb
C
Tumor Draining Lymph Nodes
Cd45+7AAD-
p = 0.25
T Cells
p = 0.39
Cd3+Cd4+ T
p = 0.57
Cd3+Cd8+ T
p = 0.39
Cd4 / Cd8 ratio
p = 0.57
Cd4+Cd25+ T
p = 0.25
Cd19+ B
p = 0.25
Cd11b+F4/80+
p = 0.79
Nk Cells
p = 0.79
Counts / Gram
Ratio
PBS huCD39 mAb
D
dLN Cd3+ T
p = 0.14
CD25 MFI
PBS huCD39 mAb
G
Body Weight
Body Weight (g)
Time (d)
PBS
H5L5 10 mg/kg
huCD39 mAb 10 mg/kg
PDL1 mAb 5 mg/kg
huCD39 mAb 10 mg/kg +
PDL1 mAb 5 mg/kg
*
*
E
PB Cd3+ T
p = 0.57
F
Tumor Cd3+Cd4+ T
p = 0.79
Tumor Cd3+Cd8+ T
p = 0.73
CD25 MFI
PBS huCD39 mAb

Figure S6. Alteration of immune cell composition after huCD39 mAb treatment in MC38 mouse model.

(A) Graphs showing numbers of various immune cells after huCD39 mAb treatment in tumor infiltrated immune cells. (B) Graphs showing proportion of various immune cells after huCD39 mAb treatment in peripheral blood. (C) Graphs showing numbers of various immune cells after huCD39 mAb treatment in tumor draining lymph nodes. Data are shown as mean ± SD. Two mice from PBS group were euthanized on Day 25 and Day 29 respectively due to excessive tumor volume. Mann-Whitney test was used. (D-F) CD25 expression was compared in T cells after huCD39 mAb treatment from tumor draining lymph nodes (D) and peripheral blood (E) and tumor tissue (F). Data are shown as mean ± SD. Mann-Whitney test was used. (G) Body weight was monitored every 2-3 days after treatment. Data are shown as mean ± SD. *, $P < 0.05$ by two-way ANOVA test, followed by Tukey's multiple comparisons test.

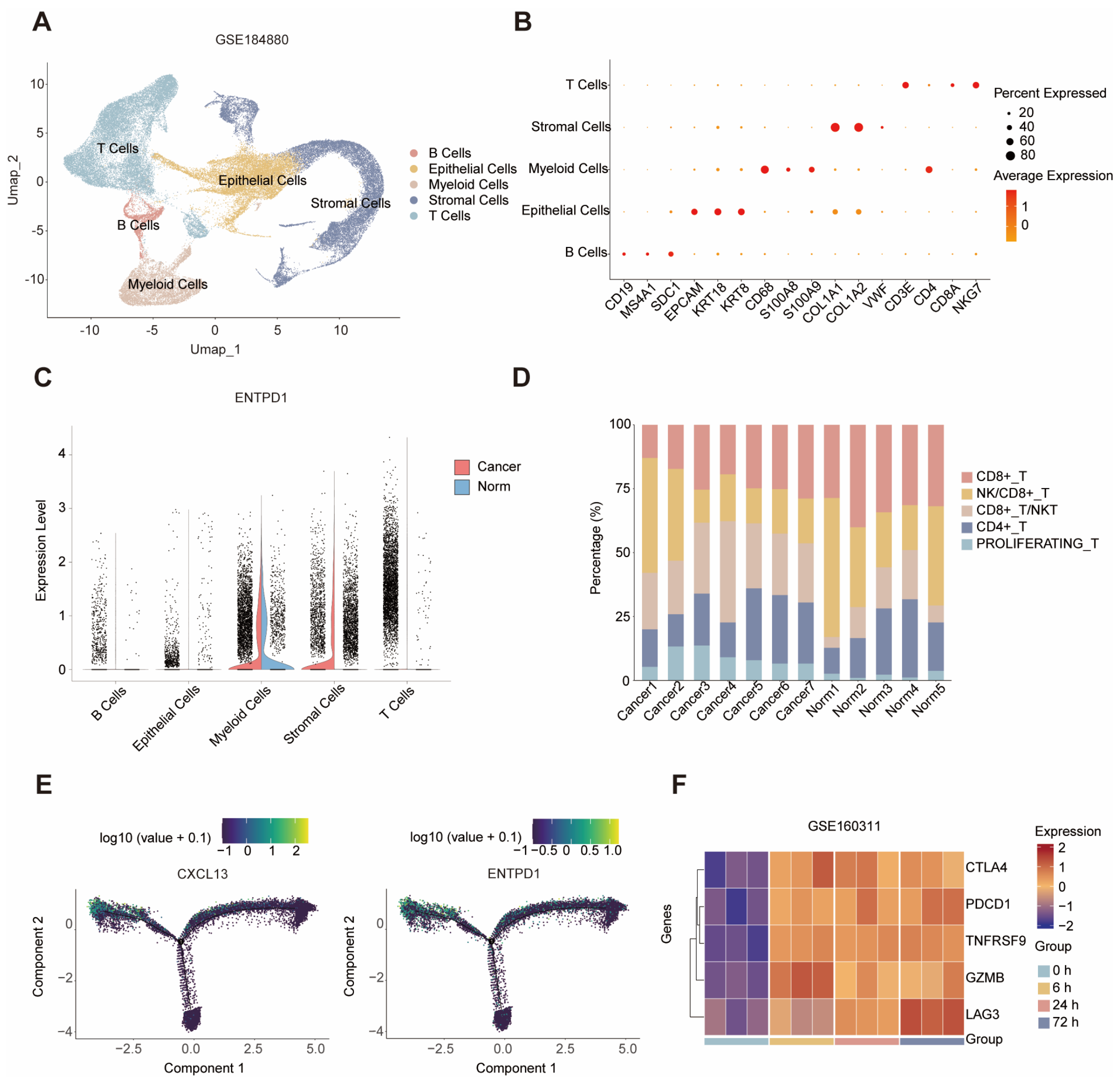

Figure S7. Bioinformatic analysis identified elevated CD39 in ovarian cancer.

(A) Umap of cells in GSE184880 ovarian cancer dataset, including 7 HGSOC samples and 5 nonmalignant ovarian samples, colored by five main clusters. (B) Dot plot showing markers in five main clusters. (C) *ENTPD1 (CD39)* expression in five main clusters from cancer and normal ovarian tissue. (D) Bar plots showing the proportion of T cell subpopulations in cancer and normal ovarian samples. (E) *CXCL13* and *ENTPD1 (CD39)* expression along pseudotime trajectory in T cell subpopulations. (F) Heat map of differential genes between tumor-specific and bystander $CD8^+$ T cells which are significant in GSE160311 dataset.

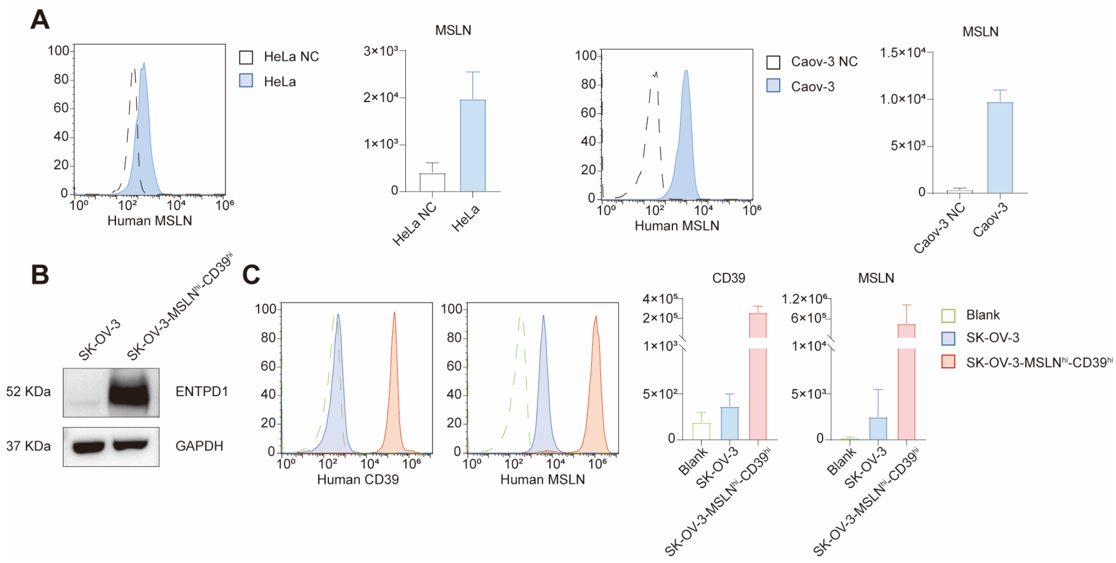

Figure S8. Expression of MSLN and CD39 in tumor cell lines.

(A) Expression of human MSLN in HeLa (left) and Caov-3 (right) was measured via flow cytometry. (B) Expression of CD39 (ENTPD1) was compared in SK-OV-3 and SK-OV-3-MSLN^{hi}-CD39^{hi} via western blotting. (C) Expression of human CD39 and human MSLN in SK-OV-3 and SK-OV-3-MSLN^{hi}-CD39^{hi} was measured via flow cytometry.

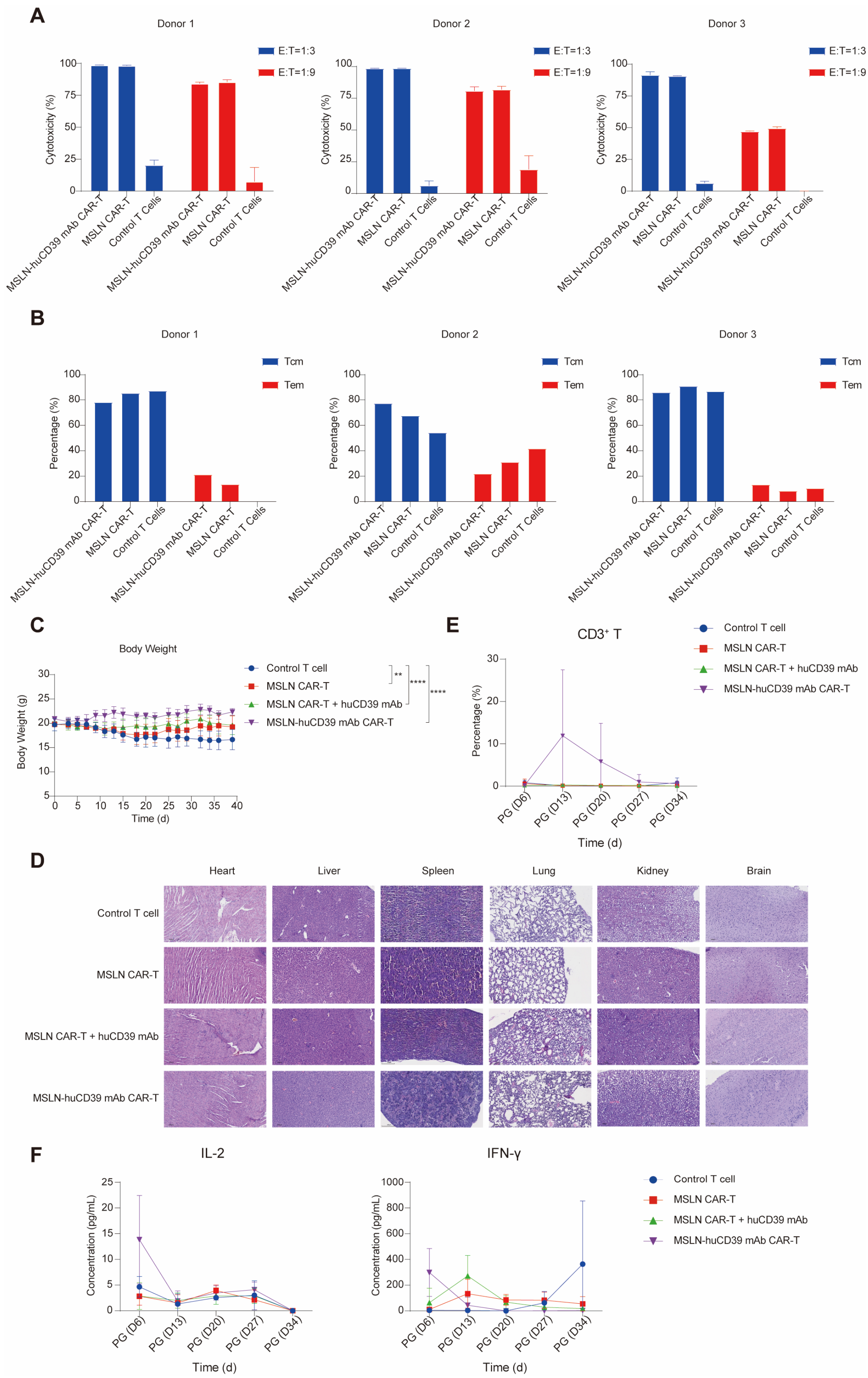

Figure S9. ***In vitro*** **and** ***in vivo*** **function of huCD39 mAb secreting MSLN CAR-T.**

(A) Cytotoxicity of MSLN CAR-T cells, MSLN-huCD39 mAb CAR-T cells and control T cells from different donors. Data are shown as mean ± SD (n = 2 technical replicates). (B) Detection of central memory T cell (Tcm) ($CCR7^{+}CD45RO^{+}$) and effector memory T cell (Tem) ($CCR7^{-}CD45RO^{+}$) proportion by flow cytometry on Day 7 after virus infection. (C) Body weight was monitored every 2-3 days after treatment. Data are shown as mean ± SD. *, $P < 0.05$ by two-way ANOVA test, followed by Tukey's multiple comparisons test. (D) HE staining of vital organs after treatment. (E-F) Weekly blood test showing changes of $CD3^{+}$ T proportion (E), IL-2 and IFN-γ levels (F) in B-NDG mice after CAR-T cells infusion via flow cytometry and Cytometric Bead Array. Data are shown as mean ± SD.

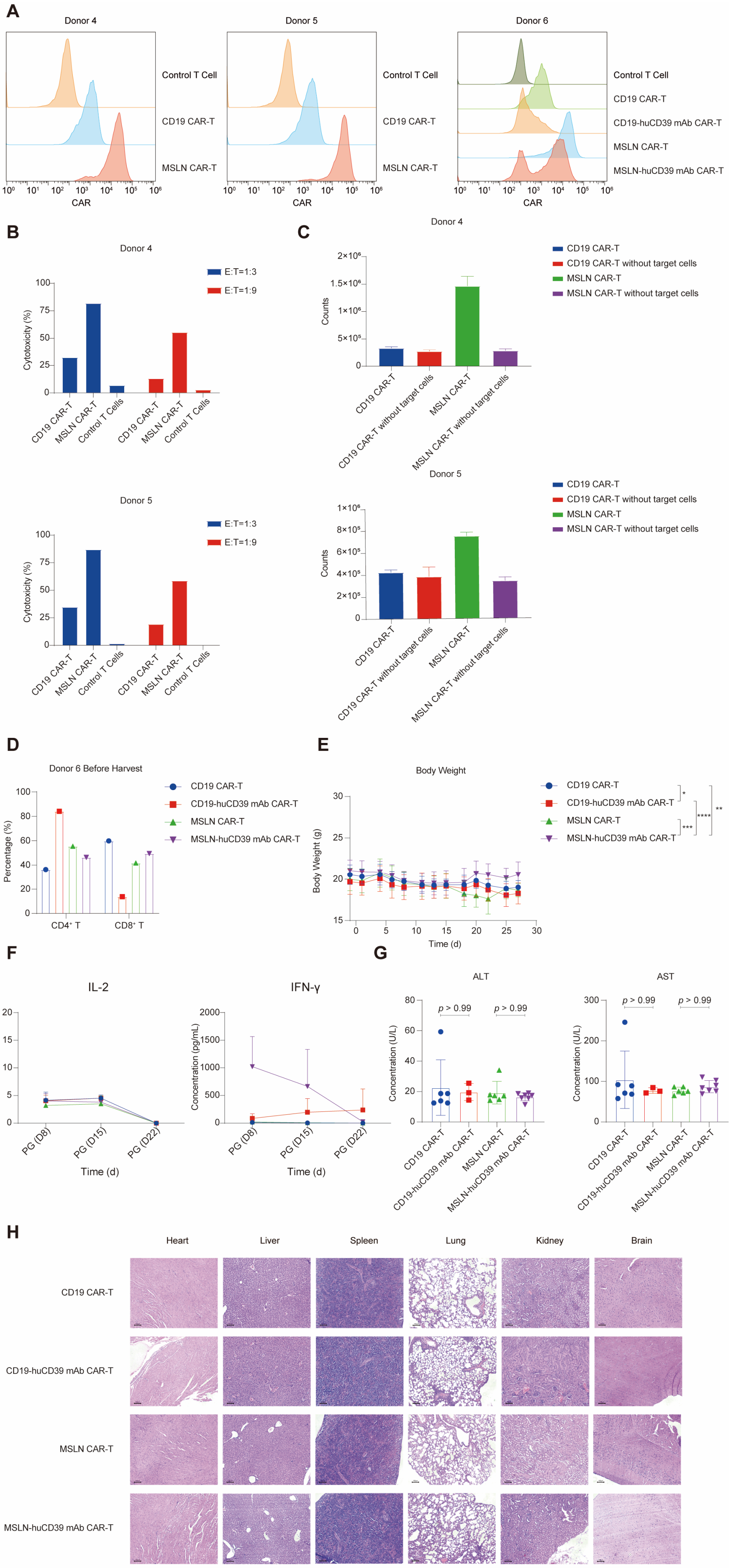
A
Donor 4
Donor 5
Donor 6
Control T Cell
CD19 CAR-T
MSLN CAR-T
CD19-huCD39 mAb CAR-T
MSLN-huCD39 mAb CAR-T
CAR
B
C
Cytotoxicity (%)
E:T=1:3
E:T=1:9
Control T Cells
Counts
CD19 CAR-T without target cells
MSLN CAR-T without target cells
D
Donor 6 Before Harvest
Percentage (%)
CD4⁺ T
CD8⁺ T
E
Body Weight
Body Weight (g)
Time (d)
F
IL-2
IFN-γ
Concentration (pg/mL)
PG (D8)
PG (D15)
PG (D22)
G
ALT
AST
Concentration (U/L)
p > 0.99
H
Heart
Liver
Spleen
Lung
Kidney
Brain

Figure S10. ***In vitro*** **and** ***in vivo*** **function of huCD39 mAb secreting CAR-T cells targeting CD19 and MSLN.**

(A) MSLN CAR-T cells and CD19 CAR-T cells were incubated with 5 mg/kg MSLN-his protein or CD19-his protein respectively, and APC-conjugated anti-his secondary antibody was applied, CAR expression was measured via flow cytometry. (B) Cytotoxicity of CD19 CAR-T cells and MSLN CAR-T cells from different donors. (C) After coculturing with SK-OV-3-MSLN^{hi}-CD39^{hi} ovarian cells, CAR^{+} T cells were numbered based on cell counting using AO/PI staining combined with flow cytometry, cumulative fold change was calculated. CAR-T cells without targeted cells were used as negative controls. Data are shown as mean ± SD. (n = 1-3 technical replicates). (D) CD4/CD8 proportion in CAR-T cells from donor 6 was detected via flow cytometry before harvest for freezing. (E) Body weight was monitored every 2-3 days after treatment. Data are shown as mean ± SD. *, $P < 0.05$; **, $P < 0.01$; ***, $P < 0.001$; ****, $P < 0.0001$ by two-way ANOVA test, followed by Tukey's multiple comparisons test. (F) Weekly blood test showing changes of IL-2 and IFN-γ levels in B-NDG mice after CAR-T infusion via Cytometric Bead Array. Data are shown as mean ± SD. (G) Alanine aminotransferase (ALT) and aspartate aminotransferase (AST) levels were tested. Data are shown as mean ± SD. One-way ANOVA test, followed by Dunn's multiple comparisons test was used. (H) HE staining of vital organs after treatment.

Table S1. EC50 of 24 tested anti-CD39 antibodies against CD39 protein via ELISA

**ID**	**EC50 (nM)**
B391-F04	0·6753
B391-A04	27·1
B391-G06	4·93
B393-D07	50·37
B39C6-G06	59·26
B39C8-G05	46·33
B391-A11	21·62
B393-G11	254·9
B395-F11	62·5
B395-G01	41·5
B39C1-B01	57·25
B39C2-E11	7·773
B391-D02	Ambiguous
B391-A01	27·03
B39C1-B03	26·76
B39C5-C04	46·32
B391-A09	48·29
B39C7-H08	8·532
B39C5-D07	Ambiguous
B39C6-F10	Ambiguous
B39C7-B04	Ambiguous
B39C7-A07	Ambiguous
B39C7-H10	7·953
B39C8-E10	24·17

Table S2. Affinity of 24 tested anti-CD39 antibodies against CD39 protein via BLI

Number	ID	Loading Sample ID	KD (M)	Kon (1/Ms)	Kdis (1/s)	Full R^2
4	B391-F04	39-4	2.553E-10	3.15E+05	8.05E-05	0·9996
5	B391-D02	B391-D02	6.586E-09	9.67E+04	6.37E-04	0·9974
6	B391-A04	B391-A04	7.758E-09	1.00E+05	7.78E-04	0·994
7	B391-G06	B391-G06	5.228E-09	8.55E+04	4.47E-04	0·9949
8	B391-A01	B391-A01	1.072E-08	8.21E+04	8.80E-04	0·9922
10	B391-A09	B391-A09	3.166E-09	9.62E+05	3.05E-03	0·9095
12	B391-A11	B391-A11	5.478E-10	2.61E+05	1.43E-04	0·9983
17	B393-D07	39-17	8.128E-10	1.65E+05	1.34E-04	0·9993
18	B393-G11	18	1.592E-10	4.49E+05	7.14E-05	0·9968
26	B395-G01	39-26	2.164E-10	1.75E+05	3.78E-05	0·999
27	B395-F11	27 B395-F11	3.348E-09	6.93E+04	2.32E-04	0·9845
38	B39C1-B01	38 B39C1-B01	3.812E-08	4.17E+03	1.59E-04	0·9465
40	B39C1-B03	40	4.862E-09	1.52E+05	7.37E-04	0·9801
53	B39C2-E11	53 B39C2-E11	1.257E-07	5.91E+03	7.44E-04	0·7733
68	B39C5-C04	CD39-68	6.95E-09	1.37E+05	9.54E-04	0·9797
71	B39C5-D07	39-71	9.544E-10	5.04E+05	4.81E-04	0·9755
78	B39C6-F10	39-78	1.08E-09	1.84E+05	1.98E-04	0·9908
80	B39C6-G06	39-80	4.094E-11	1.75E+05	7.18E-06	0·9987
81	B39C7-B04	39-81	8.917E-10	5.53E+05	4.93E-04	0·9549
82	B39C7-H08	CD39-82	8.159E-10	4.89E+05	3.99E-04	0·9618
83	B39C7-H10	39-83	2.559E-09	3.70E+05	9.46E-04	0·9635
84	B39C8-E10	39-84	2.487E-10	8.97E+05	2.23E-04	0·9045
86	B39C8-G05	39-86	1.407E-10	2.13E+05	2.99E-05	0·9991
88	B39C7-A07	39-88	6.269E-10	8.18E+05	5.13E-04	0·9282
